# Computational Network Pharmacology–Based Strategy to Capture Key Functional Components and Decode the Mechanism of Chai-Hu-Shu-Gan-San in Treating Depression

**DOI:** 10.3389/fphar.2021.782060

**Published:** 2021-11-12

**Authors:** Kexin Wang, Kai Li, Yupeng Chen, Genxia Wei, Hailang Yu, Yi Li, Wei Meng, Handuo Wang, Li Gao, Aiping Lu, Junxiang Peng, Daogang Guan

**Affiliations:** ^1^ National Key Clinical Specialty/Engineering Technology Research Center of Education Ministry of China, Guangdong Provincial Key Laboratory on Brain Function Repair and Regeneration, Neurosurgery Institute, Department of Neurosurgery, Zhujiang Hospital, Southern Medical University, Guangzhou, Guangzhou, China; ^2^ Institute of Integrated Bioinformedicine and Translational Science, Hong Kong Baptist University, Hong Kong, China; ^3^ Department of Biochemistry and Molecular Biology, School of Basic Medical Sciences, Southern Medical University, Guangzhou, China; ^4^ Department of Neurosurgery, Nanfang Hospital, Southern Medical University, Guangzhou, China; ^5^ Huiqiao Medical Center, Nanfang Hospital, Southern Medical University, Guangzhou, China; ^6^ Department of Radiology, Nanfang Hospital, Southern Medical University, Guangzhou, China; ^7^ Modern Research Center for Traditional Chinese Medicine, Shanxi University, Taiyuan, China; ^8^ Guangdong Key Laboratory of Single Cell Technology and Application, Southern Medical University, Guangzhou, China

**Keywords:** Chai-Hu-Shu-Gan-San, depression, network pharmacology model, effect propagation space, intervention-response proteins, contribution index

## Abstract

Traditional Chinese medicine (TCM) usually plays therapeutic roles on complex diseases in the form of formulas. However, the multicomponent and multitarget characteristics of formulas bring great challenges to the mechanism analysis and secondary development of TCM in treating complex diseases. Modern bioinformatics provides a new opportunity for the optimization of TCM formulas. In this report, a new bioinformatics analysis of a computational network pharmacology model was designed, which takes Chai-Hu-Shu-Gan-San (CHSGS) treatment of depression as the case. In this model, effective intervention space was constructed to depict the core network of the intervention effect transferred from component targets to pathogenic genes based on a novel node importance calculation method. The intervention-response proteins were selected from the effective intervention space, and the core group of functional components (CGFC) was selected based on these intervention-response proteins. Results show that the enriched pathways and GO terms of intervention-response proteins in effective intervention space could cover 95.3 and 95.7% of the common pathways and GO terms that respond to the major functional therapeutic effects. Additionally, 71 components from 1,012 components were predicted as CGFC, the targets of CGFC enriched in 174 pathways which cover the 86.19% enriched pathways of pathogenic genes. Based on the CGFC, two major mechanism chains were inferred and validated. Finally, the core components in CGFC were evaluated by *in vitro* experiments. These results indicate that the proposed model with good accuracy in screening the CGFC and inferring potential mechanisms in the formula of TCM, which provides reference for the optimization and mechanism analysis of the formula in TCM.

## Introduction

Depression belongs to the mental health disorder, which is an emotional disorder that causes persistent sadness and loss of interest, and is the leading cause of worldwide disability (Malhi and Mann, 2018). Previous reports have estimated that one in six people will develop the disorder during their lifetime (Kessler et al., 2005). Many studies show that depression may be associated with genetic, environmental, psychological factors, and environmental factors (Virtanen et al., 2019). Currently, western medicine mainly adopts selective serotonin reuptake inhibitor (SSRI) (Bondar et al., 2020), serotonin norepinephrine reuptake inhibitor (SNRI) (Bondar et al., 2020), norepinephrine, noradrenergic and specific serotonergic antidepressants (NaSSA), tricyclic antidepressants, and monoamine oxidase inhibitor (MAOI) for treating depression (Delgado and Moreno, 2000; Narasingam et al., 2017). However, western medicine, as the mainstream drug for treating depression, has a single mechanism of action, which leads to certain side effects and drug resistance. Traditional Chinese medicine (TCM), as a new antidepressant, can make up for the deficiency of western medicine because of its multicomponent, multitarget, and multi-mechanism pharmacological mechanism, with relatively small side effects and can be used for a long time (Shi et al., 2019; Zong et al., 2019; Ren et al., 2021).

Chai-Hu-Shu-Gan-San (CHSGS) is comprised of seven botanical drugs and were extracted with water solution: *Bupleurum scorzonerifolium* Willd. (*Bupleuri Radix*, Chaihu) (6 g), *Citrus reticulata* Blanco (*Pericarpium Citri Tangerinae*, Chenpi) (6 g), *Ligusticum striatum* DC. (*Rhizoma Ligustici Chuanxiong*, Chuanxiong) (4.5 g), *Cyperus rotundus* L. (*Rhizoma Cyperi*, Xiangfu) (4.5 g), *Citrus × aurantium* L. (*Fructus Aurantii*, Zhiqiao) (4.5 g), *Paeonia lactiflora* Pall. (*Radix Paeoniae*, Shaoyao) (4.5 g), *and Glycyrrhiza uralensis* Fisch. ex DC. (*Glycyrrhrizae Radix*, Gancao) (1.5 g)*.* CHSGS has been widely applied in treating depression and has achieved remarkable results (Meng et al., 2018; Wang et al., 2019a; Huang et al., 2019). Previous pharmacological studies have indicated that CHSGS treatment markedly prevented the ethological changes in the chronic variable stress (CVS)–induced depression rat model, including the open-field test, body weight changes, and sucrose preference test (Su et al., 2011). It has been found that CHSGS treatment can alleviate depression behavior by improving sugar water consumption and the ERK1/2 mRNA expression in the hippocampus of chronic unpredictable mild stress (CUMS) depression model rats (Wang et al., 2011). In addition, the pharmacological experimental study has found that orally administered CHSGS to depression mice models had higher SOD and catalase CAT activities, lower malondialdehyde MDA values, and higher glutathione GSH levels compared with those of the mice in the model group, suggesting that antioxidant activity of CHSGS should make contributions to its antidepression effect (Li et al., 2010). These experimental pharmacology results showed that CHSGS had evident beneficial effects in treating depression.

TCM usually plays therapeutic roles in the form of formulas in treating complex diseases. The formula has a multicomponent and multitarget mode of action during the therapy process, and these components and targets constitute the all-to-all effect network of TCM formulas in treating diseases. In the treatment procedure, some components in the effect network have auxiliary function, while others have major therapeutic actions, which were defined as the core group of functional components (CGFC). It refers to the components with suitable pharmacological features and closely associated with the effectual response to diseases. Detecting the CGFC that takes fundamental function in treating complex diseases is a big challenge due to the incomplete comprehending of the complex mechanism of multicomponents and multitargets in TCM. Optimizing formulas and obtaining CGFC are the key steps to reduce components with side effects or without activity and analyze the treatment mechanism of Chinese botanical drug formulas. Several network pharmacology–based formula optimization models have been proposed. However, these models mainly focus on the analysis of the component–target network and ignore the construction of the effect propagation space which links the drug targets to the pathogenic genes (Lee et al., 2018; Wang et al., 2018; Li et al., 2019). Studies showed that the components of Chinese medicine could play pharmacological roles through protein–protein interactions (PPI), which means the therapeutic effect of components in TCM can be transmitted through the PPI network (Chen and Cui, 2017; Gan et al., 2018; Guo et al., 2019b). Thus, it is reasonable to design a strategy to capture the CGFC based on component analysis, target prediction, and effect propagation space construction.

Currently, a new system pharmacology strategy was developed to capture the CGFC and clarify the molecular mechanisms of CHSGS in treating depression. The potential pathogenic genes of depression were extracted by analyzing the literature reports and published databases. All components of CHSGS were obtained from the database and literature and were further screened to obtain the potential active components. Three prediction tools were utilized to predict the targets of these active components. And then, the potential pathogenic genes and active component–target networks were utilized to establish effective intervention space to identify the intervention-response proteins. The intervention-response proteins selected from the effective intervention space were utilized to screen the CGFC by using the cumulative contribution coefficient (CCC) module. The CGFC was utilized to speculate the mechanisms of CHSGS in the therapy of depression.

## Methods

### Pathogenic Genes Collection and Protein–Protein Interaction Data Integration

Genes related to depression reported in DisGeNET (Pinero et al., 2017), GeneCards (Safran et al., 2010), and OMIM (Amberger et al., 2015) databases were extracted, and the number of published reports was recorded as the number of evidence, which was used to indicate the correlation between a gene and depression. The comprehensive PPI data were downloaded and integrated from Dip (Salwinski et al., 2004), HPRD (Keshava Prasad et al., 2009), Intact (Kerrien et al., 2012), Mint (Licata et al., 2012), BioGRID (Oughtred et al., 2019), and STRING (Szklarczyk et al., 2019), which were used for mapping the pathogenic genes and targets of active components.

### Collect Chemical Components of CHSGS

All chemical components of CHSGS were extracted from the TCMSP Database (Ru et al., 2014) (http://lsp.nwsuaf.edu.cn/tcmsp.php), TCM@Taiwan (Chen, 2011) (http://tcm.cmu.edu.tw/zh-tw), TCMID (Huang et al., 2018b) (http://www.megabionet.org/tcmid/), SymMap (Wu et al., 2019) (https://www.symmap.org/), and ETCM (Xu et al., 2019) (http://www.nrc.ac.cn:9090/ETCM/index.php/Home/Index/index.html). The concentration of chemical components of the botanical drugs in CHSGS was extracted from the published reports (Supplementary Table S2). The Open Babel toolkit (version 2.4.1) was employed to convert the chemical structure of components to the canonical SMILES format for further analysis (O’Boyle et al., 2011).

### Select Potential Active Components of CHSGS Based on ADMET Models

Nine ADME models, including Lipinski’s rules of five Daina et al., 2017, oral bioavailability (OB (%F)), GI absorption Daina and Zoete, 2016, human Ether-à-go-go-Related Gene (hERG) inhibition, and carcinogenicity evaluation of components were utilized to select the active components from CHSGS. The Lipinski’s rules specifically includes molecular weight <500 Da, number of donor hydrogen bonds <5, number of acceptor hydrogen bonds <10, −2 <the log P < 5, and meets only the criteria of 10 or fewer rotatable bonds. Components that met OB ≥ 30% were kept as candidate components. The screening criterion of GI absorption was defined as high. The hERG inhibition was calculated by a toxicity model which was proposed in the PreADMET webserver (Lee et al., 2012); components with a high level of hERG inhibition were removed. Here the carcinogenicity of each component in CHSGS was evaluated by the PreADMET webserver (Lee et al., 2012); the components with the negative feature of carcinogenicity were kept for further analysis.

### Targets Prediction

Similarity Ensemble Approach (SEA) (Keiser et al., 2007), HitPick (Liu et al., 2013), and SwissTargetPrediction (Daina et al., 2019) were utilized to predict the targets of active components in CHSGS. The Open Babel toolkit (version 2.4.1) was employed to convert canonical SMILES.

### Network Construction

The component–target (C-T) networks of CHSGS were established by utilizing Cytoscape software (Version 3.7.0) (Shannon et al., 2003). NetworkAnalyzer (Assenov et al., 2008) was utilized to analysis the topological parameters of networks.

### Construct the Effective Intervention Space and Evaluate the Intervention-Response Proteins

Constructing effect intervention space from complex networks acted by TCM can retain highly correlated small molecular targets and pathogenic genes to the greatest extent. We map the component–target network to the PPI network which integrated from Biogrid, String, DIP, HPRD, INTACT, and MINT, and then, map the pathogenic genes with the literature support to the PPI network to establish the component–target–pathogenic gene–disease network. The importance of nodes in the network is the main basis for analyzing the key components of the network.

There are many reported methods for calculating node importance, such as degree, closeness centrality, betweenness centrality, clustering coefficient, neighborhood connectivity, and average shortest path length. Here, we design a new method to characterize the importance of nodes; we defined $Net_{ctpd}=\left\{N,E\right\}$ , where *N* means nodes which represent components, targets, pathogenic genes, and disease. *E* means edges which represent component–target–pathogenic gene–disease interactions:

$$\emptyset =\underset{i<n}{\max}\left(d_{1\to 2},d_{1\to 3},d_{1\to 4}\cdots d_{i\to j}\cdots d_{\left(\frac{\left(n\left(n-1\right)\right)}{2}-1\right)\to \left(\frac{\left(n\left(n-1\right)\right)}{2}\right)}\right)$$

$$IM_{i}=\sqrt{\frac{\left(\emptyset +1\right)-\sum d_{jk}\left(i\right)/m}{\emptyset }\times \frac{\sum_{j}^{n}\sum_{k}^{n}g_{jk}\left(i\right)/g_{jk}}{n\left(n-1\right)/2}}$$

$$IM_{median}=median\left\{IM_{1},IM_{2},IM_{3},\cdots ,IM_{n}\right\}$$

$$EIS=\overset{n}{\underset{i=1}{\cup }}IM_{\left(Net_{ctpd}\right)i}>IM_{median}$$

$IM_{i}$ means the significance of node *i* in the network; The ∅ is the largest distance between two nodes in the network. If a network is disconnected, ∅ is the maximum distance among all the connected components. $g_{jk}$ represent the number of paths between node *j* and *k*. $g_{jk}\left(i\right)$ is the number of paths from node *j* to node k and through node *i*. $d_{jk}\left(i\right)$ is the number of shortest paths from node j to node k and through node i; m is the number of total shortest paths in the whole network which pass the node *i*; *n* means the total number of nodes in the network. EIS represent the effective intervention space. The nodes in the effective intervention space were identified as intervention-response proteins. And then, we performed functional pathway analysis using intervention-response proteins and depression pathogenic genes to evaluate whether the intervention-response proteins could cover the pathogenic genes of depression at the functional level.

### Develop CCC Model to Select CGFC

The CGFC is hidden in the components of the effective intervention space. We define the network coverage of each component *i* in the effective intervention space as $w_{i}$ . The contribution rate of targeted to pathogenic genes is $v_{i}$ . The maximum expected network coverage rate of CGFC is R. In these variables, $R>0,w_{i}>0,v_{i}>0,1\leq i\leq n$ , GCFC is required to be found from *n* components, so that the cumulative contribution rate of targeted to pathogenic genes is the largest. The specific calculation formula is as follows:

$$\mathrm{CCC}=\max\sum_{i=1}^{n}v_{i}x_{i}$$

$$\sum_{i=1}^{n}w_{i}x_{i}\leq Rx_{i}\in \left\{0,1\right\},1\leq i\leq n$$

Therapeutic Mechanisms of CHSGS for Depression

Set the subproblems of the given question as:

$${\mathrm{CCC}}_{sub}=\max\sum_{k=1}^{n}v_{k}x_{k}$$

$$\sum_{k=1}^{n}w_{k}x_{k}\leq R_{sub}x_{k}\in \left\{0,1\right\},1\leq k\leq n$$

$m\left(i,C_{sub}\right)$ is the optimal solution when the expected network coverage is $R_{sub}$ , and the optional component is *y*. From the optimal substructure properties, the recursive formula for calculating $m\left(i,C_{sub}\right)$ can be established as follows:

$$m\left(i,R_{sub}\right)=\{\begin{array}{cc} \max\left\{m\left(i+1,R_{sub}\right),m\left(i+1,R_{sub}-w_{i}\right)+v_{i}\right\} & R_{sub}\geq w_{i}\\ m\left(i+1,R_{sub}\right) & 0\leq R_{sub}<w_{i} \end{array}$$

$$m\left(n,R_{sub}\right)=\{\begin{array}{cc} v_{n} & R_{sub}\geq w_{n}\\ 0 & 0\leq j<w_{n} \end{array}$$

### Gene Ontology and Pathway Analysis

The clusterProfiler (Yu et al., 2012) package of R software was utilized to perform Gene Ontology (GO) analysis. The Kyoto Encyclopedia of Genes and Genomes (KEGG) database (Kanehisa and Goto, 2000) was employed to perform KEGG pathway enrichment analyses. The *p*-values of GO and KEGG analyses were set at 0.05 as the cut-off criterion. The ggplot2 package was used to create graphs in the R statistical programming language (version 3.4.2). The above results were annotated by Pathview (Luo and Brouwer, 2013) in the R Bioconductor package (https://www.bioconductor.org/).

### Maximum Targeting Weight Model Calculation

We defined G=(V,E) as a weighted directed graph; V and E represents the set of set of proteins and relationships in the integrated pathway. TG and PG represent the set of target genes and pathogenic genes, respectively. For each protein pairs  (s↔t) , we use the Dijkstra method to calculate the shortest distance between them directly in the integration pathway. The maximum target weight score can be calculated as follows:

$$Score\;\left(s↔t\right)=\{\begin{array}{c} \sum_{i=1}^{n}\left(IM_{i}+R_{i}+D_{i}+R_{avg}+D_{avg}\right)\;Note_{i}\in TG,Note_{i}\in PG\\ \sum_{i=1}^{n}\left(IM_{i}+R_{i}+D_{i}+R_{avg}-D_{avg}\right)\;Note_{i}\notin TG,Note_{i}\in PG\\ \sum_{i=1}^{n}\left(IM_{i}+R_{i}+D_{i}-R_{avg}+D_{avg}\right)\;Note_{i}\in TG,Note_{i}\notin PG\\ \sum_{i=1}^{n}\left(IM_{i}+R_{i}+D_{i}-R_{avg}-D_{avg}\right)\;Note_{i}\notin TG,Note_{i}\notin PG \end{array}$$

$IM_{i}$ , $R_{i}$ , and $D_{i}$ indicate the topological importance, strength of documentary evidence, and strength of regulated by multiple components of node *i*, respectively. $R_{avg}$ and $D_{avg}$ means the average strength of documentary evidence of all nodes that represent pathogenic genes and the average strength of nodes that represent target genes which are regulated by multiple components. The $IM_{i}$ can be calculated by our proposed methods, the $R_{i}$ and $D_{i}$ were calculated by $\frac{NV-\min\left(PG\right)}{\max\left(PG\right)-\min\left(PG\right)}$ and $\frac{NC-\min\left(TG\right)}{\max\left(TG\right)-\min\left(TG\right)}$ , NV means the evidence number of one node, min(PG) and max(PG) means the minimum and maximum number of document supports in pathogenic genes. NC represent the number of components that regulates a node, min(TG) and max(TG) means minimum and maximum number of compounds which could regulate the node.

### Experimental Validation

#### Reagents

Vanillic acid (≧98% purity by HPLC) was obtained from Jingzhu Biotechnology Co., Ltd. (Nanjing, China), and corticosterone (purity ≧98%) was purchased from TCI Shanghai (Shanghai, China). RPMI-1640 medium, fetal bovine serum (FBS), 0.25% trypsin, and 3-(4,5-dimethylthiazol-2-yl)-2,5-diphenyltetrazolium bromide (MTT) were purchased from Shanghai Sangon Biotechnology Co. Ltd. (Shanghai, China). Poly-L-polylysine (PLL) was purchased from Sigma-Aldrich Co. (St. Louis, United States). The commercial kit for measuring the lactate dehydrogenase (LDH) was purchased from Nanjing Jiancheng Bioengineering Co., Ltd. (Nanjing, China).

#### Cell Culture and Treatment

The differentiated PC12 cells were purchased from the Cell Bank of the Chinese Academy of Sciences (Shanghai, China). Cells were cultured in an incubator at 37°C with an RPMI-1640 medium containing 10% FBS. When the cells reached 80% confluency, the cells were treated with vanillic acid for 3 h; then, the cells were treated with corticosterone (400 μM).

#### Cell Viability Assay

PC12 cells (2 × 10^4^ per/well) were seeded in 96-well plates, which were coated with PLL (0.01%). After 24 h incubation, PC12 cells were treated with 0.1, 1, 10, 25, 50, and 100 μM vanillic acid and corticosterone. MTT was superinduced to a 96-well plate for 4 h; then, the culture supernatant was removed. Finally, DMSO was utilized to dissolve the purple crystals. The plate reader was utilized to detect the absorbance at 570 nm.

#### Measurement of LDH Release

The release of LDH was detected by utilizing the assay kits according to specifications.

### Statistical Analysis

All data were expressed as mean ± SEM. The differences were analyzed by one-way ANOVA for multiple comparisons. Results were considered as statistically significant if the *p*-value was <0.05.

## Results

**FIGURE 1:**
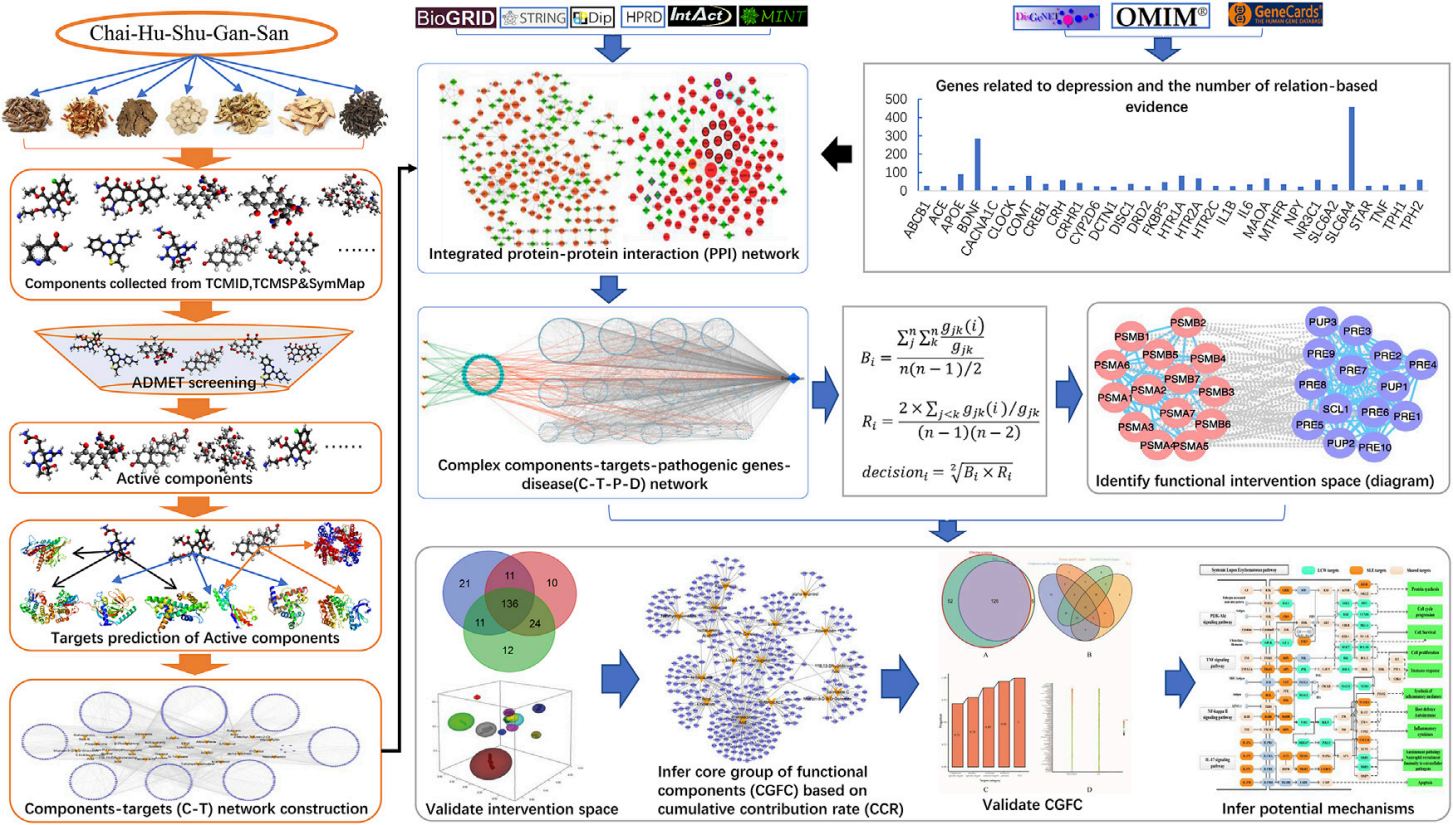
The work scheme of our network pharmacology approach. Firstly, the pathogenic genes of depression, components of CHSGS and protein–protein interactions (PPI) were extracted from the published literature reports and databases. Previously proposed ADMET models were used to select potential active components. Targets of these active components were predicted to establish the C-T network. Then, the pathogenic genes with frequency of evidence and active components-targets network were mapped to the integrated PPI to construct components-targets-pathogenic genes-disease (CTPD) network. This complex network contains a large amount of redundant information. In order to obtain the most useful treatment information, find out the parts with greater contribution to intervention and have higher correlation between targets of active components, we developed a new node importance characterization method. Based on this method, we select the effective intervention space from the CTPD network. In the effective intervention space, we predicted CGFC by using the CCC model. Finally, the CGFC was used to infer the underlying molecular mechanism of CHSGS in treating depression.

A new bioinformatics analysis of the network pharmacology model was designed to investigate the core functional components of CHSGS in treating depression and to speculate its potential mechanism of action (Figure 1).

### Extraction and Analysis of the Pathogenic Genes of Depression

**FIGURE 2:**
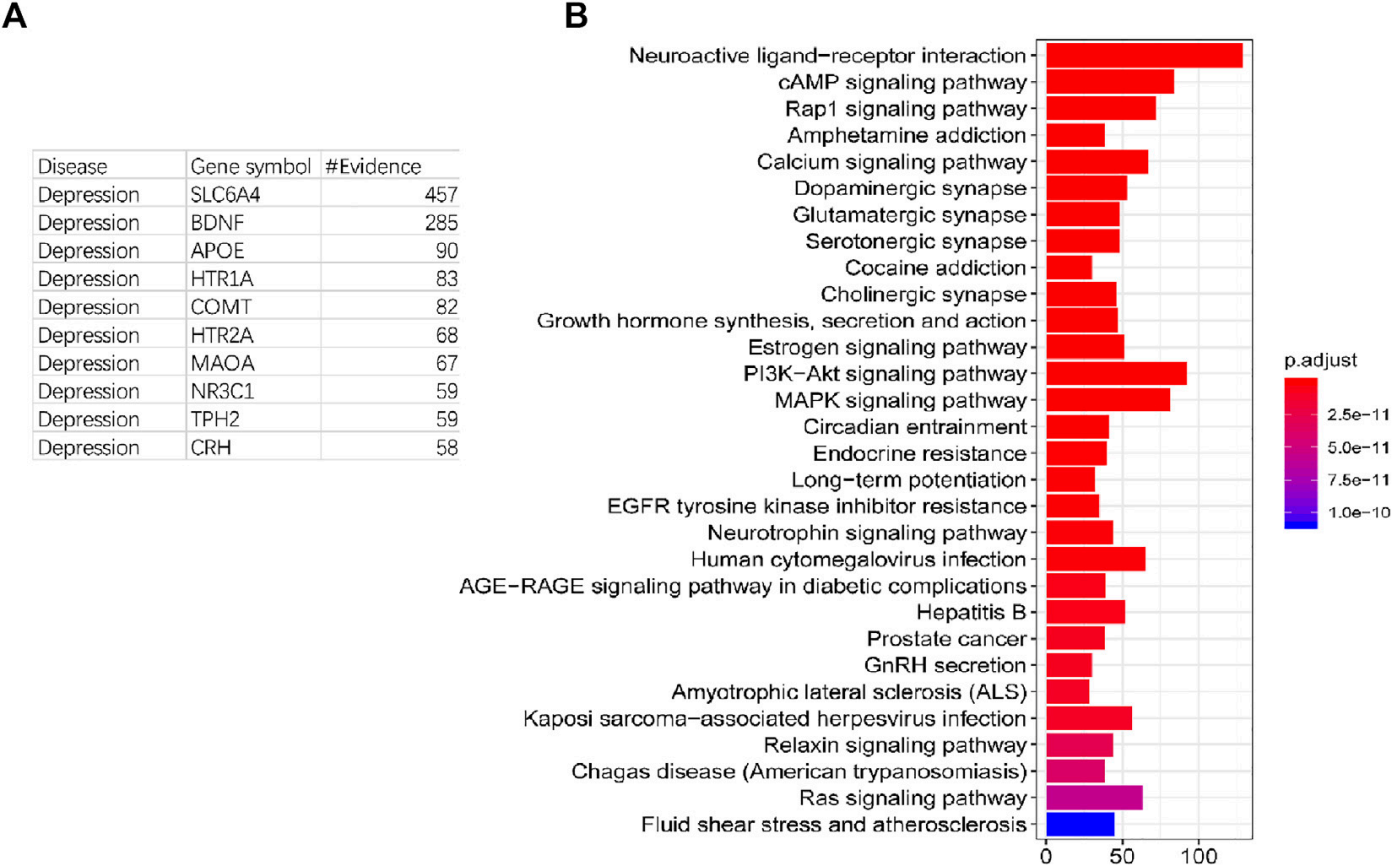
Number of evidence and function analysis of pathogenic genes. **(A)**: The list of top 10 genes with the highest number of evidences. **(B)**: Top 30 enriched pathways of pathogenic genes.

The process of depression is related to an intricate series of changes in gene expressions and phenotypes. These different phenotypic changes are accompanied by a large number of gene expression changes, which could be marked as the pathogenic genes both at the diagnosis and intervention level. In order to extract comprehensive pathogenic genes with confirmed evidence of depression, the DisGeNET and OMIM databases were used to collect the genes associated with depression. 1,329 genes and 3,069 documentary evidences have been reserved as pathogenic genes with confirmed evidence for further construction of the effective intervention space (Supplementary Table S1). Most of these pathogenic genes have less documentary evidence, 882 genes have only one documentary evidence (Table 1), and 42 genes have more than 15 documentary supports. In order to test whether genes with more documentary support have more extensive functions, we performed KEGG and GO analyses on all pathogenic genes and found that genes with more literature support are associated with more pathways and GO terms. Genes with more than or equal to 15 and less than 20 literature support have the largest average pathways and GO term association numbers (Table 1). The genes with the top 10 literature reports are SLC6A4, BDNF, APOE, HTR1A, COMT, HTR2A, MAOA, NR3C1, TPH2, and CRH (Figure 2A). These genes are mainly related to depression in single nucleotide polymorphism and expression regulation. Among the top 30 enriched pathways the neuroactive ligand–receiver interaction, dopaminergic synapse, MAPK signaling pathway, and PI3k-akt signaling pathway have been widely reported to be related to depression (Figure 2B).

**TABLE 1:** Analysis of documentary evidence of the pathogenic genes of depression.

Interval of literature support number	Number of genes distributed in literature support	Average number of pathways related to each pathogenic gene	Average number of GO terms related to each pathogenic gene
1–2	882	0.21	3.77
2–3	191	0.92	13.46
3–5	93	1.85	26.52
5–10	96	1.74	27.17
10–15	25	2.16	55.64
15–20	11	9.73	78.18
20–40	19	6.16	75.37
Over 40	12	2.25	69.08

### Chemical Analysis of CHSGS

Chemical analysis exerts pivotal effects for clarifying substance basis and the molecular mechanism of formulas. The concentration of specific chemical components in CHSGS was captured by extracting from the published reports (Supplementary Table S2). The concentration of components in botanical drugs from chemical analysis provides a reliable basis for optimizing active components.

### Select Active Components in CHSGS

A total of 1,012 components from seven botanical drugs in CHSGS were extracted from TCM@Taiwan, TCMID, TCMSP, SymMap, and ETCM databases (Supplementary Table S3). Previously proposed ADME screening methods were applied to capture potential active components. After ADME screening, 249 active components in CHSGS passed the combined filtering criteria which were integrated by Lipinski’s rule, OB, GI, hERG, and carcinogenicity (Table 2, Supplementary Table S4).

**TABLE 2:** The number of components collected in the published databases and active components screened by ADMET models in CHSGS.

Botanical drugs	#Components	#Active components
*Bupleurum scorzonerifolium* Willd. (CH)	354	79
*Citrus reticulata* Blanco (CP)	74	14
*Ligusticum striatum* DC. (CX)	193	35
*Cyperus rotundus* L. (XF)	104	29
*Citrus × aurantium* L. (ZQ)	17	6
*Paeonia lactiflora* Pall. (SY)	166	45
*Glycyrrhiza uralensis* Fisch. ex DC. (GC)	283	82
Total (remove duplication)	1,191 (1,012)	290 (249)

**FIGURE 3:**
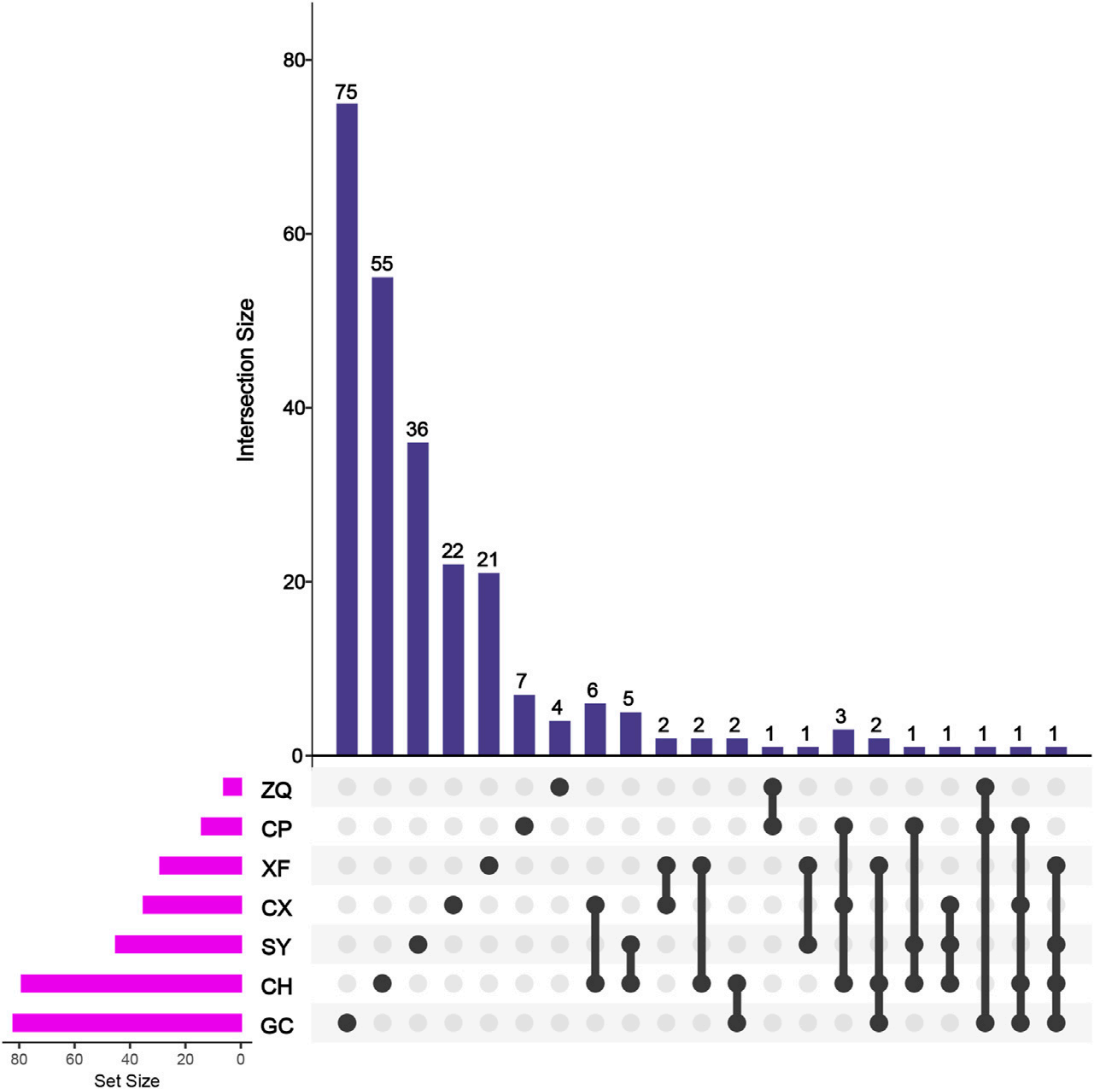
Specific and common components of botanical drugs in CHSGS.

By analyzing these active components, we found that 29 of them were shared by two or more botanical drugs (Figure 3). Kaempferol (CHSGS20) was a common component in CH, XF, SY, and GC. Vanillin (CHSGS26) was another common component in CH, CP, and CX. Naringenin was shared by CH, ZQ, and GC.

Except the shared components, most of the botanical drugs play therapeutic roles through their specific ingredients. *Bupleurum scorzonerifolium* Willd. (CH), *Citrus reticulata* Blanco (CP), *Ligusticum striatum* DC. (CX), *Cyperus rotundus* L. (XF), *Citrus × aurantium* L. (ZQ), *Paeonia lactiflora* Pall. (SY), and *Glycyrrhiza uralensis* Fisch. ex DC. (GC) have 55, 7, 22, 21, 4, 36, and 75 specific active components, respectively. These results show that different components in CHSGS act as a synergistic mode; in this process, the specific components play a leading role.

### Predict Targets of Active Components and Establish the Components-Targets Network

We used three tools to predict the target genes of the active components and got 1,286 predicted target genes. To probe the therapeutic mechanism of CHSGS in treating depression, 249 active components and 1,286 targets (Supplementary Table S5) were utilized to establish the C-T network. Most of these active components are associated with multiple targets, resulting in 9822 component–target associations between active components and targets. The average number of targets of per component is 39.39. It indicates the multicomponent and multitarget features of CHSGS for treating of depression. Among these components, vanillic acid (CHGSG4, degree = 258) has the highest number of targets.

Moreover, we previously described that the common components shared by two or more botanical drugs and specific components of certain botanical drugs, kaempferol (CHSGS20), vanillin (CHSGS26), naringenin (CHSGS137), limetin (CHSGS158), L-Menthone (CHSGS35), and patchouli alcohol (CHSGS29) also have higher targets number. The degrees of these components are 78, 95, 36, 61, 16, and 16, respectively. These results suggested that the pivotal roles of these components in treating depression and further confirmed that CHSGS has the effect of multi-targets treatment of depression.

In the component–target network, the mean degree of targets for different components is 7.63. Interestingly, majority of these targets are confirmed related to the pathogenesis of depression and that may indicate potential therapeutic mechanisms of CHSGS on depression. These results indicated that CHSGS act synergistically to treat depression in a multi-component manner.

### Intervention-Response Proteins Selection and Validation From Effective Intervention Space

**FIGURE 4:**
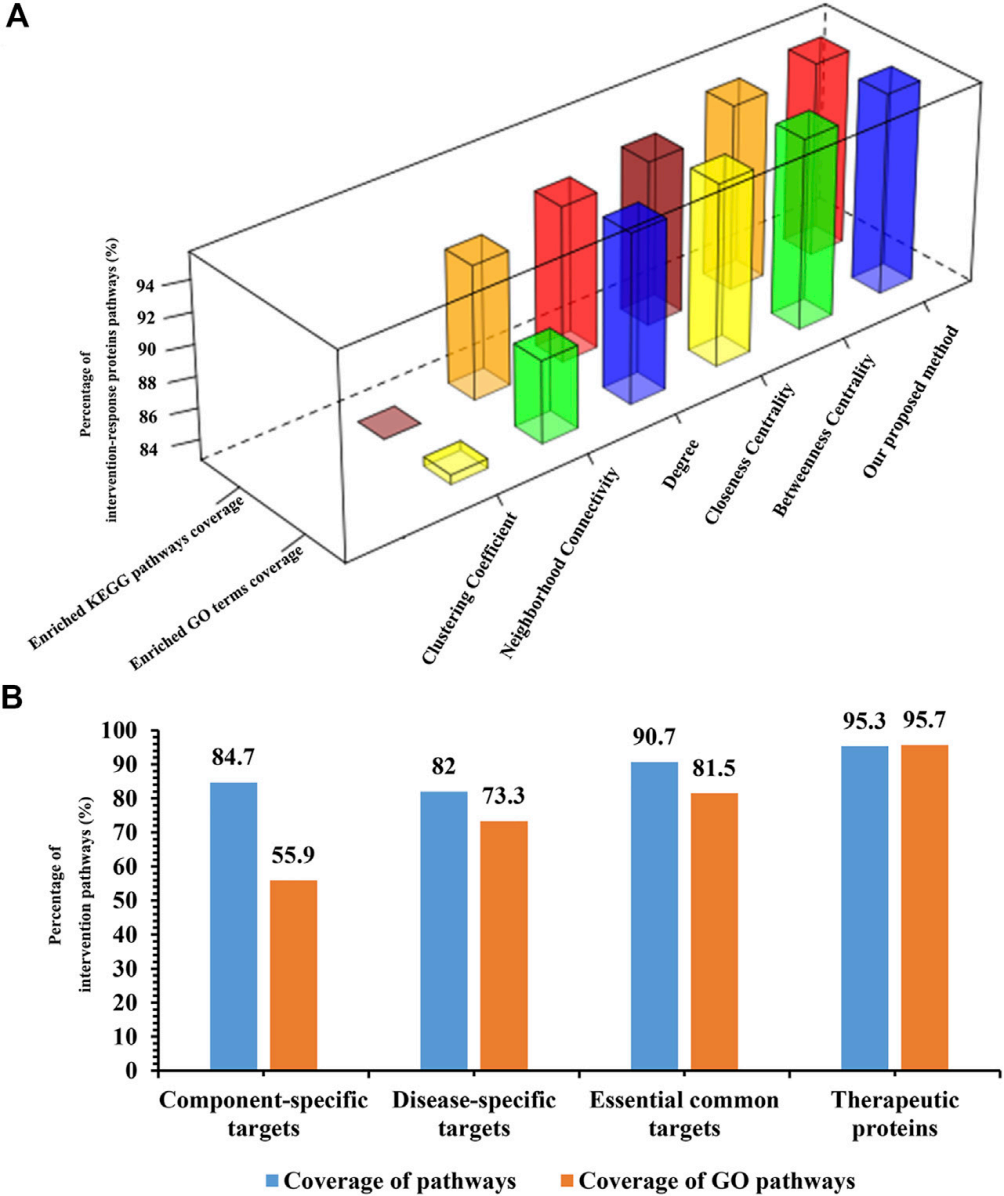
Validation of effective intervention space. **(A)**: Compare our proposed methods with other widely used methods. **(B)**: The proportion histogram of component-specific targets, disease-specific targets, essential common targets, and therapeutic proteins in common enriched pathways and GO terms.

The process of drug action is a complex process which is responded by series of different proteins or genes. These proteins and genes are regulated by or co-occurred with other proteins or genes in the process of disease occurrence and development, which constitute a complex network related to disease progression. The therapeutic effect could be propagated through the network. Here, we map the pathogenic genes and targets of C-T network to the integrated PPI network to construct the component–target–pathogenic gene–disease (CTPD) network. This complex network contains 2,548 nodes and 41,378 edges. In this huge network, the correlation and transmission of target genes to pathogenic genes is the fundamental part. Thus, we take targets–pathogenic genes (T-P) subnetwork in the CTPD network for further analysis. Importance of nodes in a network is the key topological property that can be used to evaluate the roles of nodes in the network. For each node *i* in the T-P network, if the important score of a node is more than the median important score of all nodes in the network, such node is believed to play a pivotal role in the network structure and can be treated as a hub node (Liu et al., 2016). Following this rule, the important score of each node in the T-P network was calculated and then compared with the median important score of all nodes in the network; the passed nodes and their edges in the T-P network were kept and defined as effective intervention space. The effective intervention space contains 1,019 nodes and 18,466 edges; each node represents one therapeutic protein, and thus, we identified 1,019 intervention-response proteins from the effective intervention space. To test whether the intervention-response proteins we selected from effective intervention space could cover the pathogenic genes of depression at the functional level, we used all target genes of active components and pathogenic genes of depression to do pathway and GO analysis and found that there are 150 and 1991 common enriched pathways and GO terms, respectively. These enriched pathways and GO terms are the functional basis of CHSGS in treating depression, which we selected for evaluating effective intervention space and intervention-response proteins. The number of intervention-response proteins enriched pathways and GO terms were 143 (*p* < 0.05) and 1905 (*p* < 0.05). The intervention-response proteins enriched pathways and GO terms were found to cover 95.3 and 95.7% of the 150 common pathways and 1991 common GO terms (Figure 4A). These results prove that we proposed effective intervention space based on the novel importance calculation method of nodes which is reliable. We compare the performance of proposed models by using the coverage of 150 common KEGG enriched pathways and 1991 common GO terms with other widely used methods on the calculation of node importance, including betweenness centrality, closeness centrality, clustering coefficient, degree, and neighborhood connectivity. Results show that our model has the highest coverage both at enriched pathways and GO terms. These results further prove the reliability and accuracy of our novel node importance calculation method.

There are three categories of intervention-response proteins in effective intervention space. The first category is the direct interactions between the component targets and pathogenic genes. We defined this category as the essential common targets. The second category is the interactions of disease-specific targets. The third category is the interactions of component-specific targets. In order to test whether the effective intervention space can be replaced by essential common targets, disease-specific targets or component-specific targets for further optimization. We performed pathway analysis on essential common targets, disease-specific targets, component-specific targets, respectively. Results show that the coverage proportion of enriched pathways of three categories compared with the enrichment pathways of pathogenic genes is 90.7, 82, and 84.7%, respectively, (Figure 4B). The coverage proportion of enriched GO terms of three categories compared with the enrichment pathways of pathogenic genes is 81.5, 73.3, and 55.9%, respectively, (Figure 4B). Far less than that of the intervention-response proteins, these results confirmed the accuracy and reliability of our proposed effective intervention space and further demonstrated that the intervention-response proteins selected in the effective intervention space play a key role in the pathogenesis of depression.

### CGFC Extracted Form Effective Intervention Space

**FIGURE 5:**
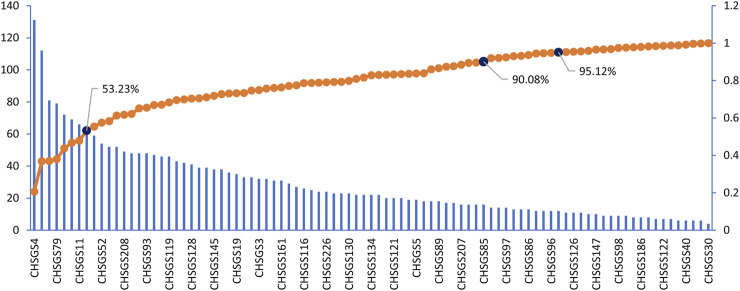
The accumulative CCC of active components in effective intervention space.

The CCC module was established to optimize effective components and get the CGFC, which would be used to clarify the molecular mechanism of CHSGS in the therapy of depression. According to the contribution accumulation results, the top eight components including vanillic acid (CHSGS4), DTR (CHSGS163), apocynin (CHSGS109), isovanillic acid (CHSGS79), phenylacetic acid (PAC, CHSGS149), Karenzu DK2 (CHSGS177), benzoic acid (BOX, CHSGS11), and quercetin (CHSGS2) contribute to 53.23% target coverage of effective proteins. For further analysis, 61 components can contribute to 90.08% target coverage of effective proteins, while the target coverage of effective proteins quickly increased to 95.12% after the 71 components were taken in the CCC model. Thus, we selected the 71 components as the CGFC (Figure 5 and Table 3). Higher target coverage of effective proteins proved that the CGFC may play the leading role and generate combination effects in the treatment of depression.

**TABLE 3:** The information of CGFC in CHSGS.

ID	molecule_name	MW	Hdon	Hacc	RBN	logP	OB (%)
CHSGS2	quercetin	302.24	5	7	1	1.07	46.43
CHSGS3	4-Hydroxybenzoic acid	138.12	2	3	1	1.58	30.15
CHSGS4	vanillic acid	168.15	2	4	2	1.7	35.47
CHSGS5	Nonanal	142.24	0	1	7	3.81	40.28
CHSGS11	benzoic acid	122.12	0	2	1	1.72	31.55
CHSGS13	Jaranol	314.29	2	6	3	2.8	50.83
CHSGS17	isorhamnetin	316.26	4	7	2	1.31	49.6
CHSGS18	formononetin	268.26	1	4	2	3.01	69.67
CHSGS19	Calycosin	284.26	2	5	2	2.82	47.75
CHSGS20	kaempferol	286.24	4	6	1	1.23	41.88
CHSGS26	vanillin	152.15	1	3	2	1.31	52
CHSGS51	Polyhydric alcohols	92.09	3	3	2	-1.93	72.87
CHSGS52	Indole-3-carboxylic acid	161.16	2	2	1	1.79	33.86
CHSGS54	adenine	135.13	3	4	0	-0.38	62.81
CHSGS56	salicylic acid	138.12	2	3	1	1.96	32.13
CHSGS60	Dibutylphenol	206.32	1	1	2	4.9	38.9
CHSGS63	Methylgallate	184.15	3	5	2	1.01	30.91
CHSGS67	albiflorin_qt	318.32	2	6	4	0.53	66.64
CHSGS68	(3R,3aR,6S,7aR)-6-hydroxy-3,6-dimethyl-3a,4,7,7a-tetrahydro-3H-benzofuran-2,5-dione	198.22	1	4	0	0.02	104.94
CHSGS76	scoparone	206.19	0	4	2	1.91	74.75
CHSGS78	hexanoic acid	116.16	1	2	4	1.88	73.08
CHSGS79	Isovanillic acid	168.15	2	4	2	1.81	39.42
CHSGS81	Valerophenone	162.23	0	1	4	2.94	42.58
CHSGS82	Isobutyrophenone	148.2	0	1	2	2.54	80.37
CHSGS85	Perlolyrine	264.28	2	3	2	2.66	65.95
CHSGS86	senkyunolide-C	204.22	1	3	2	3.49	46.8
CHSGS87	senkyunolide-E	204.22	1	3	2	1.93	34.4
CHSGS89	1-Acetyl-beta-carboline	210.23	1	2	1	2.53	67.12
CHSGS93	WLN: 2VR	134.18	0	1	2	2.15	60.17
CHSGS96	3-cyclohexen-1-ol	98.14	1	1	0	0.96	70.57
CHSGS97	4,7-Dihydroxy-3-butylphthalide	222.24	2	4	3	2.69	106.09
CHSGS109	Apocynin	166.17	1	3	2	1.62	31.71
CHSGS114	Chryseriol	300.26	3	6	2	2.53	35.85
CHSGS116	m-Methylacetophenone	134.18	0	1	1	2.08	40.63
CHSGS118	Lupiwighteone	338.35	3	5	3	3.23	51.64
CHSGS119	7-Methoxy-2-methyl isoflavone	266.29	0	3	2	3.48	42.56
CHSGS121	Visnagin	230.22	0	4	1	1.92	44.25
CHSGS128	Isodalbergin	268.26	1	4	2	3.76	35.45
CHSGS130	Khell	260.24	0	5	2	1.78	33.19
CHSGS134	Hyndarin	355.43	0	5	4	3.09	73.94
CHSGS141	Nonenoic acid	156.22	1	2	6	3.53	65.17
CHSGS143	3,5,6,7-tetramethoxy-2-(3,4,5-trimethoxyphenyl)chromone	432.42	0	9	8	2.59	31.97
CHSGS145	Ayapanin	176.17	0	3	1	2.06	41.55
CHSGS146	8-NONENOIC ACID	156.22	1	2	7	2.84	52.31
CHSGS149	Phenylacetic acid	136.15	1	2	2	1.72	72.35
CHSGS151	cis-2-Undecenal	168.28	0	1	8	4.93	47.07
CHSGS154	Ethyl protocatechuate	182.17	2	4	3	1.83	35.77
CHSGS160	(E)-non-2-en-4-one	140.22	0	1	5	2.98	37.78
CHSGS161	Cumic acid	164.2	1	2	2	2.86	45.78
CHSGS163	(2R)-2-amino-3-(1H-indol-3-yl)propionic acid	204.23	4	3	3	-1.1	75.63
CHSGS164	Veratryl alcohol	168.19	1	3	3	1.27	71.49
CHSGS171	(E)-1-(2,4-dihydroxyphenyl)-3-(2,2-dimethylchromen-6-yl)prop-2-en-1-one	322.35	2	4	3	4.46	39.62
CHSGS177	Karenzu DK2	224.25	0	2	4	3.14	62.26
CHSGS181	Gancaonin B	368.38	3	6	4	3.14	48.79
CHSGS184	2-(3,4-dihydroxyphenyl)-5,7-dihydroxy-6-(3-methylbut-2-enyl)chromone	354.35	4	6	3	2.99	44.15
CHSGS201	(-)-Medicocarpin	432.42	4	9	4	1.26	40.99
CHSGS206	3-(4-hydroxyphenyl)-7-methoxychromen-4-one	268.26	1	4	2	2.92	38.37
CHSGS207	1-Methoxyphaseollidin	354.4	2	5	3	3.66	69.98
CHSGS208	Quercetin der.	330.29	3	7	3	2.55	46.45
CHSGS209	(Z)-1-(2,4-dihydroxyphenyl)-3-phenylprop-2-en-1-one	240.25	2	3	3	3.3	73.18
CHSGS211	3′-Methoxyglabridin	354.4	2	5	2	3.76	46.16
CHSGS223	*Glycyrrhiza* flavonol A	370.35	4	7	1	2.18	41.28
CHSGS226	Phaseol	336.34	2	5	2	4.59	78.77
CHSGS227	Mipax	194.18	0	4	4	1.96	57.4
CHSGS228	5,7-dihydroxy-2-(3-hydroxy-4-methoxyphenyl)chroman-4-one	302.28	3	6	2	2.52	47.74
CHSGS234	Citromitin	404.41	0	8	7	2.61	86.9
CHSGS237	nobiletin	402.39	0	8	7	2.61	61.67
CHSGS238	7-Demethylsuberosin	230.26	1	3	2	3.41	41.19
CHSGS241	N-Methyltyramine	151.21	2	2	3	0.48	75.52
CHSGS245	Nerylacetone	194.31	0	1	6	4.59	45.53
CHSGS247	Cubebin	356.37	1	6	4	2.54	57.13

**FIGURE 6:**
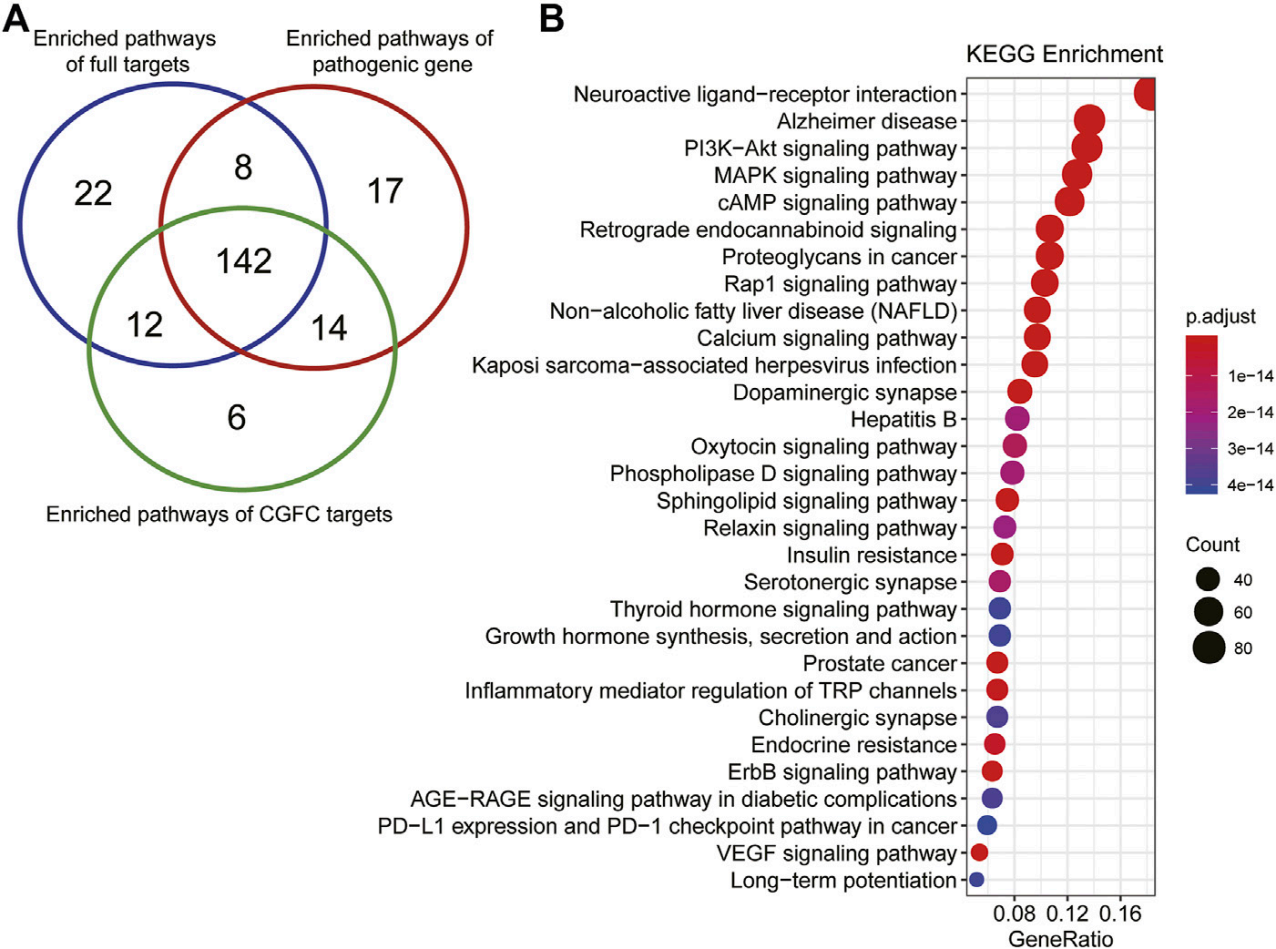
Pathway enrichment analysis of the targets of CGFC. **(A)**. Venn diagram shows the coincidence of enrichment pathways of full targets, pathogenic genes, and CGFC targets. **(B)**. The top 30 enriched pathways of CGFC targets, the size of the circle represents the number of genes enriched in the pathways, and the color change of the circle represents the significance of the enrichment of genes in the pathways.

For the analysis of CHSGS in the treatment of depression at the functional level, we performed pathway analysis using CGFC targets and depression pathogenic genes, respectively. Among them, the number of CGFC target–enriched pathways was 174 (*p* < 0.05), and the number of pathogenic gene–enriched pathways was 181 (*p* < 0.05). The CGFC target–enriched pathways were found to cover 86.19% of the pathogenic gene–enriched pathways (Figure 6A). To our surprise, the number of enriched pathways of full target is 184 (*p* < 0.05), compared to the pathogenic genes, the coverage of enriched pathway on CGFC targets and full targets was 83.7 and 81.5%, respectively. This result indicates that the CGFC selection model could effectively select key targets and remove noise. These major targets of CGFC were frequently involved in the PI3K-Akt signaling pathway (hsa04151), MAPK signaling pathway (hsa04010), cAMP signaling pathway (hsa04024), Rap1 signaling pathway (hsa04015), calcium signaling pathway (hsa04020), oxytocin signaling pathway (hsa04921), phospholipase D signaling pathway (hsa04072), sphingolipid signaling pathway (hsa04071), relaxin signaling pathway (hsa04926), thyroid hormone signaling pathway (hsa04919), ErbB signaling pathway (hsa04012), and VEGF signaling pathway (hsa04370), etc. (Figure 6B). Among these pathways, the PI3K-Akt signaling pathway (hsa04151), MAPK signaling pathway (hsa04010), and cAMP signaling pathway (hsa04024) were widely reported to be related to the onset and treatment of depression. These results demonstrate that CHSGS can exert a therapeutic role in the treatment of depression through cooperation of multi-signaling pathways.

### Potential Mechanisms Analysis of CGFC Treats Depression

**FIGURE 7:**
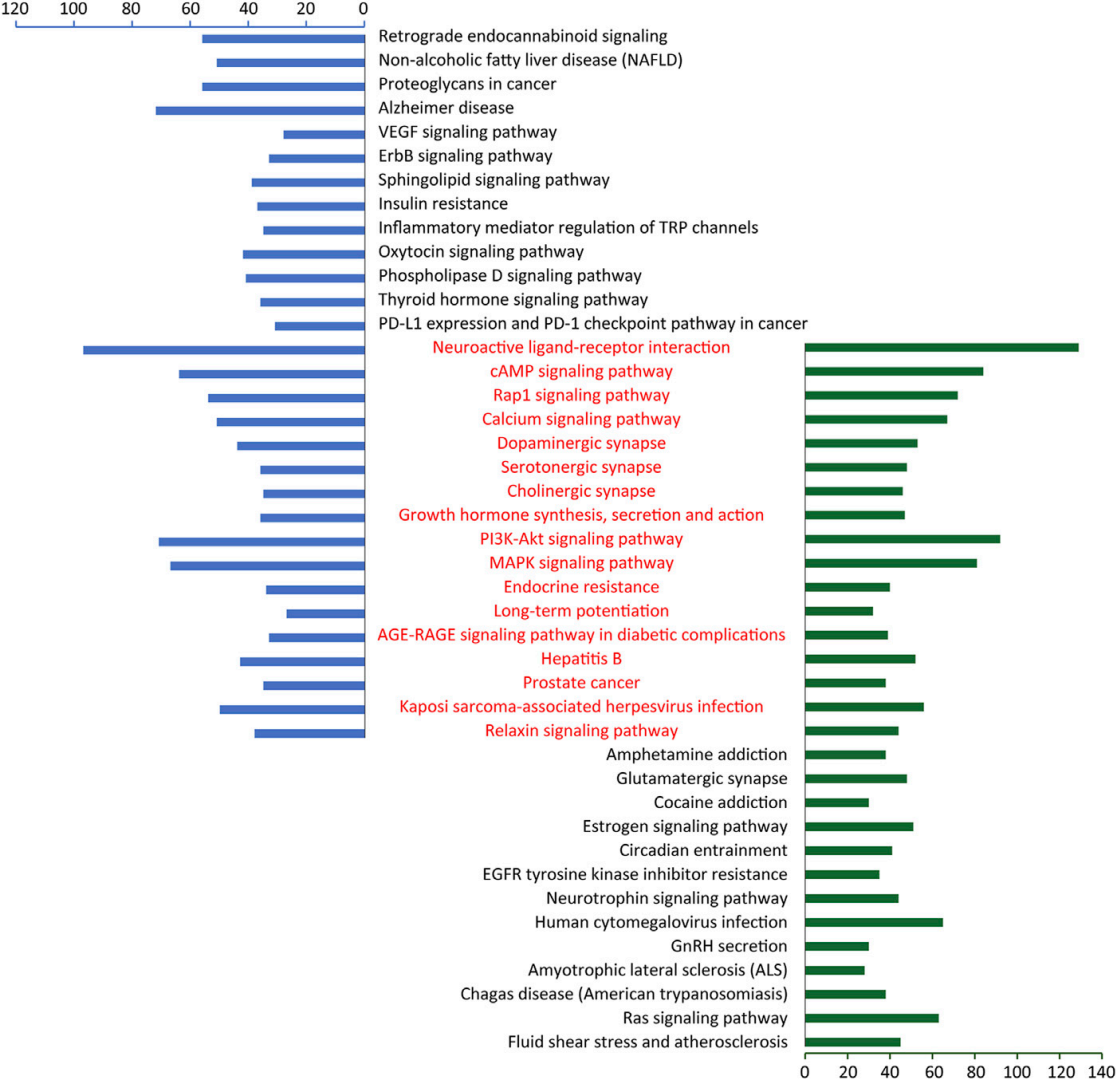
Enriched pathways of CGFC targets and pathogenic genes. The red part represents the shared pathways of CGFC targets and pathogenic genes.

In order to further clarify the potential mechanism of CGFC-mediated CHSGS in treating depression, we compared and analyzed the pathways enriched by CGFC targets and pathogenic genes and found that 17 of the top 30 pathways were overlapped (Figure 7), and among these 17 pathways, the cAMP signaling pathway (HSA 04024), dopaminergic synapse (HSA 04728), PI3K-Akt signaling pathway (HSA 04151), and MAPK signaling pathway (HSA 04010) are widely reported to be related to the pathogenesis and treatment of depression. For exploring the mechanism of CHSGS in treating depression at the system level, we constructed a comprehensive signaling pathway using these four important molecular pathways (Supplementary Figure S1).

In this comprehensive pathway, we use the maximum targeting weight (MTW) model to calculate the cascade pathways, which indicate drug enter cells through extracellular receptors to cause a cascade effect of downstream genes. 13 Cascade pathways with scores greater than 0.7 were retained for further analysis (Table 4).

**TABLE 4:** Cascade pathways predicted by the MTW model.

Potential mechanism chain	Score
ADCY1--cAMP--PRKACA--RHOA	0.962
ADCY1--cAMP--PRKACA--GRIA1	0.939
ADCY1--cAMP--PRKACA--GRIN2A	0.916
DRD1--GNAQ--PLCB1--DAG--PRKCA--FOS	0.867
ADCY1--cAMP--PRKACA--GRIA1--GSK3A	0.801
DRD5--GNAQ--PLCB1--DAG--PRKCA--FOS	0.793
ADCY1--cAMP--PRKACA--CREB3--BDNF	0.790
ADCY1--cAMP--PRKACA--CREB3--FOS	0.754
ADCYAP1R1--GNAS--ADCY1--cAMP--PRKACA--RHOA	0.735
ADCYAP1R1--GNAS--ADCY1--cAMP--PRKACA--GRIA1	0.720
ADCY1--cAMP--PRKACA--MAPK14--ATF2	0.710
ADCY1--cAMP--RAPGEF3--MAPK8--JUND	0.710
ADCYAP1R1--GNAS--ADCY1--cAMP--PRKACA--GRIN2A	0.705

**FIGURE 8:**
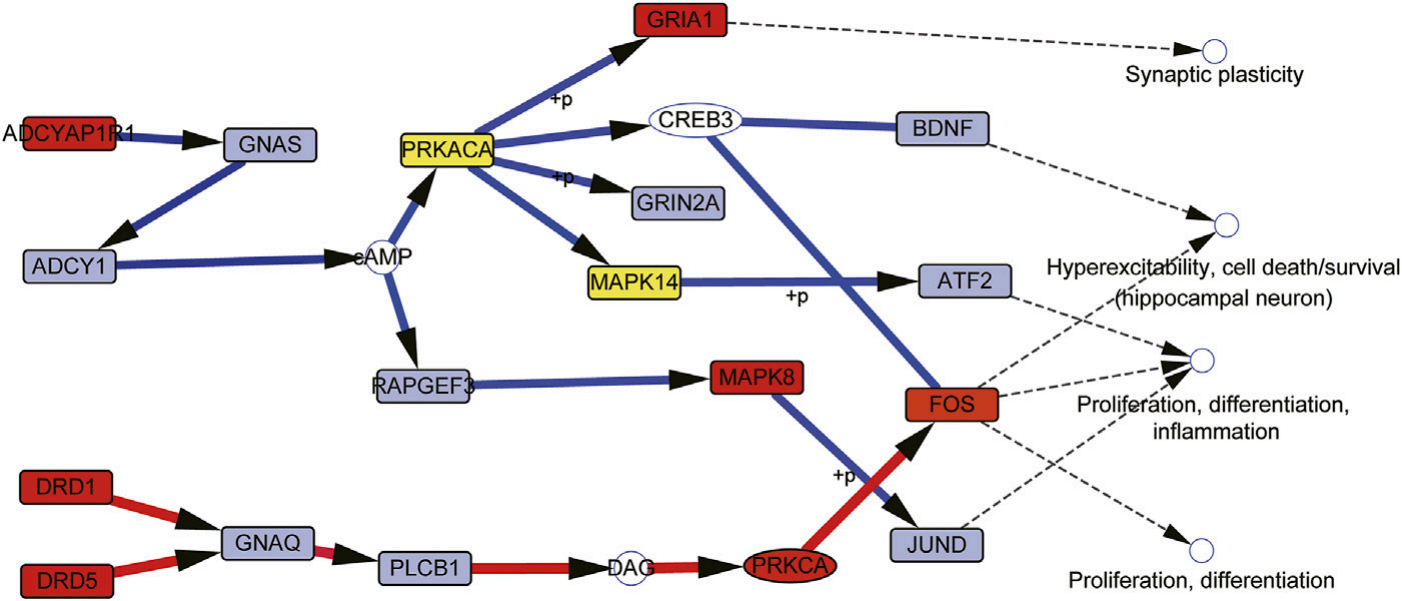
Two cascade targeting modules merged by MTW-predicted cascade pathways. Red nodes mean the genes are both the targets of CGFC and pathogenetic genes of depression. Light green nodes mean the CGFC specific target genes. Gray nodes represent the specific pathogenetic genes.

These cascade pathways are integrated, two main cascade targeting modules can be obtained (Figure 8). The first module controls the downstream GRIA1, GRIN2A, GSK3A, CREB3, BDNF, FOS, ATF2, MAPK8, MAPK14, JUND, RHOA, and other genes to treat depression through ADCYAP1R1--GNAS--ADCY1—Camp--PRKACA/RAPGEF3 cascade reactions. In this module, ADCYAP1R1, PRKACA, MAPK8, MAPK14, GRIA1, and FOS are both targeted genes of CGFC and pathogenic genes of depression; RHOA is a specific targeted gene of CGFC, and most of these genes are related to the pathogenesis or treatment of depression. The second module control downstream FOS to treat depression by targeting the DRD1/DRD5--GNAQ--PLCB1--DAG--PRKCA cascade reaction. In this module, DRD1, DRD5, PRKCA, and FOS are both pathogenic genes and targeted genes of CGFC. Most of these targeted genes are related to the pathogenesis or treatment of depression. The above results show that the components in CGFC play a therapeutic role in treating depression through a synergistic way.

### Experimental Evaluation of Important CGFC

**FIGURE 9:**
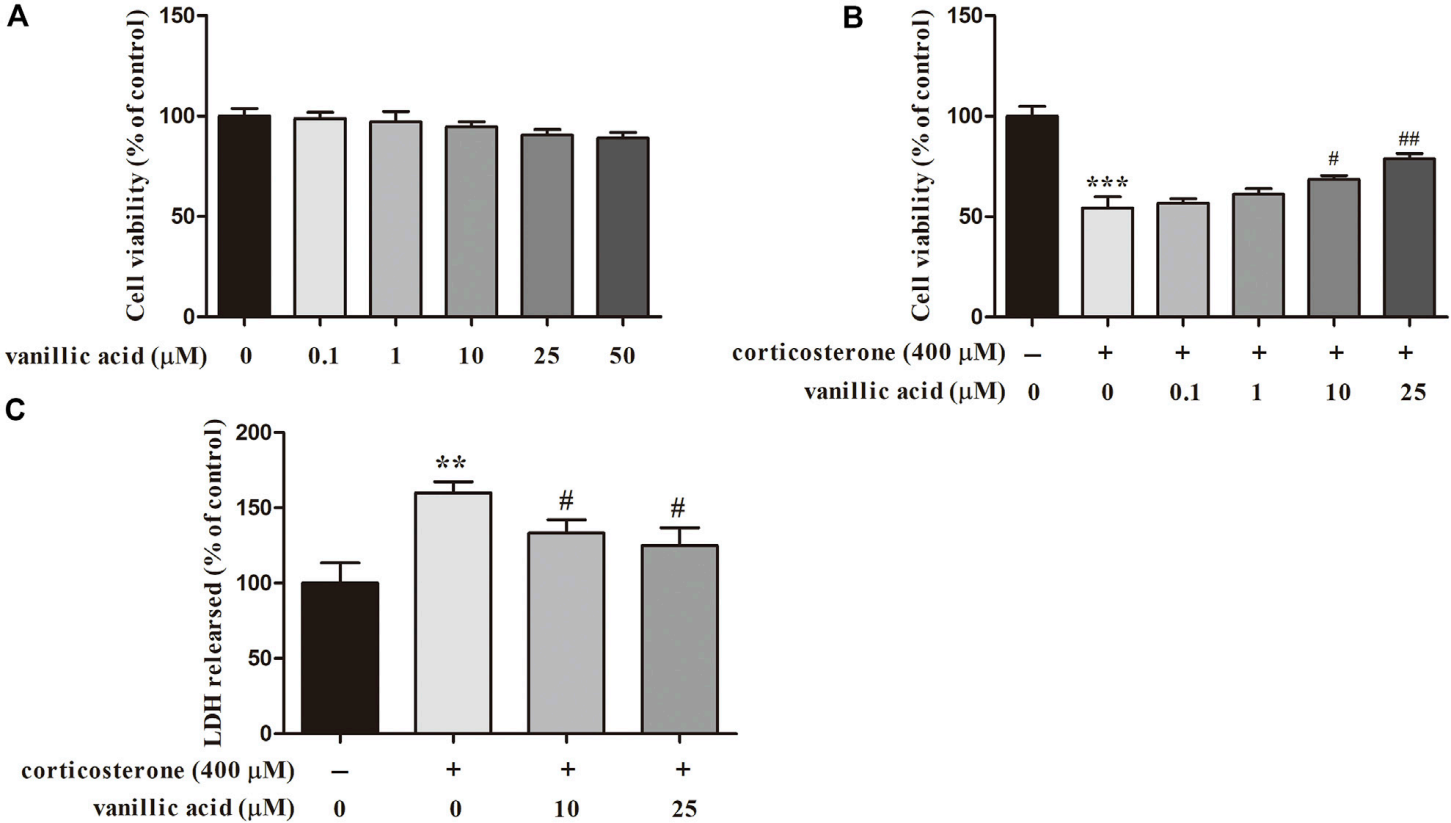
Effect of vanillic acid alone on PC12 cells **(A)** and effect of vanillic acid on corticosterone-induced apoptosis in PC12 cells **(B)**, effect of vanillic acid on LDH leakage on corticosterone-treated PC12 cells **(C)**. ^**^ *p* < 0.01, ^***^ *p* < 0.001 compared with that of the control group. ^#^ *p* < 0.05, ^##^ *p* < 0.01 compared with the corticosterone group.

To validate the accuracy and reliability of our model, the important CGFC predicted were experimentally validated in PC12 cells. The effect of vanillic acid was evaluated in PC12 cells. The results showed that 0.1, 1, 10, 25, and 50 μM vanillic acid had almost no effects to PC12 cells (Figure 9A). MTT results showed that vanillic acid (10 and 25 μM) markedly increased the cell viability as compared with that of the corticosterone group (Figure 9B). LDH was utilized to evaluate the damage and toxicity of cells. As shown in Figure 9C, the level of LDH release was markedly increased after corticosterone treatment, and vanillic acid (10 and 25 μM) significantly decreased the level of LDH, which suggested that vanillic acid possesses a protective effect against corticosterone-induced neurotoxicity in PC12 cells by reducing LDH release.

Previous pharmacological studies have shown that vanillic acid treatment could block oxidative damage in PC12 cells and exert the effect of neuroprotective (Kim et al., 2007). It has been reported that vanillic acid could improve the nervous behavior of the depressed model mice and raise the content of 5-HT in the mouse plasma, which may affect the metabolization of the 5-HT loop and the activities in the hippocampus, amygdala and other brain areas to prevent depression (Wang et al., 2013). The above experimental results suggested that vanillic acid may possess obvious beneficial effects in the treatment of depression.

## Discussion

TCM plays therapeutic roles in treating complex diseases with the characteristics of multi components and multi targets. These components and targets form a complex intervention network. How to find the most effective intervention relationship in this intervention network and find CGFC is the key to understand the material basis and molecular mechanism of TCM and is also the basis for the secondary development of TCM. At present, the main purpose of formulas optimizing based on network pharmacology is to improve the curative effect of the formula and reduce the nonpharmacological factors. According to the principle of compatibility of TCM, each formula consists of several botanical drugs, each of which contains hundreds of chemical components. Whether botanical drugs or ingredients in the formula are necessary, especially when treating a specific disease, still needs analysis and verification. Through compound optimization, these botanical drugs and components with a better intervention effect can be screened out, while those botanical drugs and components with the antagonistic effect and even side effects are removed, making the compound simpler and more effective (Wang et al., 2020a; Wang et al., 2020b; Gao et al., 2020; Yang et al., 2021).

In order to better optimize the classical formulas with clinical efficacy, network pharmacology methods and bioinformatics algorithms are used to screen the key corresponding relationships from component targets to pathogenic genes. In this process, we established a new node importance calculation method. Based on this method, we constructed the effective intervention space and traced the CGFC from the effective intervention space, then inferred the possible mechanism using the maximum target weight model based on the CGFC. It provides methodological reference for the secondary development of TCM and the development of new drugs.

At present, how to optimize and obtain the CGFC and analyze their mechanism of action is the basis for quantification of TCM. TCM emphasizes a holistic and systematic view and regards the integrated treatment of different botanical drugs and ingredients as a coordinated whole. Network pharmacology has the characteristics of systematisms and integrity and conforms to the core theory of TCM. Network pharmacology emphasizes multitarget regulation of signal pathways to improve drug efficacy and reduce toxic and side effects. Network pharmacology is widely used to speculate the potential mechanism in treating complex diseases in TCM (Wang et al., 2021). For example, to determine the potential mechanism of the formula in TCM for treating complex diseases and infer the mechanism of “treating the same disease with different methods” and “treating different diseases with the same method,” but there are few reports on optimization research of TCM based on network pharmacology. To address this issue, we proposed a novel bioinformatics analysis of the network pharmacology model to obtain the CGFC of CHSGS in the treatment of depression and analyze the potential mechanism of CGFC, which were verified by published literature reports. Our method has several advantages:

In the process of analyzing the therapeutic mechanism, network pharmacology formed a fixed analysis rule. In this rule, the first step is to collect the components of botanical drugs, do ADME/T screening for selecting the active components, then predict targets and infer the molecular mechanism. The flow chart really solves the molecular mechanism of some formulas for treating complex diseases in TCM, such as decoding the synergistic mechanism of zhi-zhu wan for functional dyspepsia (Wang et al., 2018), analyzing the underlying mechanism of Kaixin powder in treating Alzheimer’s disease (Luo et al., 2020), investigating the mechanism of Oryeong-san formula for the treatment of hypertension (Kim et al., 2019). However, there are also exist two problems. One is the redundancy and interference of component target networks. The other is that most network pharmacological analysis ignores that the intervention effect of components is a cascade transmission process, specifically refers to the transmission of the intervention effect from target genes to pathogenic genes. In order to solve these two problems, we have adopted some new strategies. The first strategy is to construct a new node importance calculation method. Comparison results show that our proposed node importance calculation method has better performance on function coverage than several commonly used node importance calculation methods. Another strategy is that we consider that the intervention effect of component targets can be transmitted to pathogenic genes through the PPI network, and based on this, we construct a complex network of components–targets–pathogenic genes–diseases, and then, use our proposed node importance calculation method to obtain the relatively important partial relationship between targets and pathogenic genes to construct and verify the effective intervention space. We found that the ratio of the EIS gene–enriched pathways and GO terms to the reference both reached above 95%, which also confirmed the reliability and accuracy of the constructed EIS. In order to further verify the EIS, we divide the relationships in the EIS into three categories. The first category is the direct interactions linking the component targets to pathogenic genes and was defined as the interactions of essential common targets. The interactions of pathogenic genes were defined as the second category. The interactions of component-specific targets belong to the third category in order to test whether the effective intervention space can be replaced by interactions of essential common targets, the interactions of pathogenic genes or the interactions of component-specific targets. In the functional pathways and GO terms enrichment analysis, we found that the genes in the effective intervention space had the highest coverage proportion compared to the enrichment pathways of pathogenic genes. This confirmed once again the accuracy and reliability of our proposed EIS and further demonstrated that the intervention-response proteins selected in the EIS play a key role in the pathogenesis of depression.

We used a dynamic programming algorithm to infer the CGFC from the genes in the effective intervention space and made functional analysis and verification. It was found that the target genes of the CGFC were enriched to 174 pathways, with 156 pathways coinciding with the enriched pathways of pathogenic genes, accounting for 86.19%, while the enriched pathways of the whole CHSGS target genes only have 150 pathways coinciding with the pathogenic genes, accounting for 82.87% of the pathogenic gene enrichment pathways. This result indicates that after optimization, invalid or weak effect relationships were removed, and the functional coverage rate was improved. It also shows the reliability of our effective intervention space and the strategy of selection of CGFC.

The CGFC contains 71 components and is extracted from the effective intervention space; after that the enrichment pathway of these component target genes is combined for decoding the potential mechanism. To address this issue, we design a maximum target weight model to make mechanism speculation. The core idea of the model is to ensure that drugs enter cells from outside and target as many pathogenic genes as possible with the lowest cost. We predicted 13 functional cascade chains and after summarizing these cascade chains. We found that they were mainly concentrated in two major cascade signal modules. The first module controls the downstream GRIA1, GRIN2A, GSK3A, CREB3, BDNF, FOS, ATF2, MAPK8, JUND and other genes to treat depression through the cascade reaction of ADCYAP1--ADCYAP1R1--GNAS--ADCY1--cAMP--PRKACA. The second module is target to the DRD1/5-GNAQ-PLC B1-DAG-PRKCA cascade signal to control the downstream FOS to treat depression. Both modules start from typical receptors and regulate downstream genes through protein kinase A or C to treat depression after cascade signal changes. Particularly, ADCYAP1R1 encodes the type I adenylate cyclase activated polypeptide receptor, which is a member of g protein coupled receptor (GPCRs). The receptor mediates various biological actions of adenylate cyclase-activated polypeptide 1 (ADCYAP1). ADCYAP1 is the main regulator of central and peripheral stress responses needed to restore and maintain internal balance (Mustafa, 2013). It can stimulate adenylate cyclase which is encoded by ADCY1 and increase cyclic adenosine monophosphate (cAMP) levels (Fimia and Sassone-Corsi, 2001; Mustafa, 2013); cAMP regulates pivotal physiologic processes including metabolism, secretion, calcium homeostasis, muscle contraction, cell fate, and gene transcription. cAMP acts directly on protein kinase A (PRKACA), PRKACA modulates, *via* phosphorylation, a number of cellular substrates, including transcription factors, ion channels, transporters, exchangers, intracellular Ca2+ -handling proteins, and the contractile machinery (Lizcano et al., 2000; Voglis and Tavernarakis, 2006; Gerlo et al., 2011). Dopamine (DA) is an important and prototypical slow neurotransmitter in the mammalian brain, where it controls a variety of functions including locomotor activity, motivation and reward, learning and memory, and endocrine regulation. Dopamine D1 and D5 receptors (DRD1 and DRD5) are typical G protein-coupled receptors (GPCR) mainly expressed in the neurogenic area, with high constitutive activity and belong to the D1-like receptors (D1Rs), D1 and D5 receptors, both positively coupled to adenylyl cyclase and cAMP production, D1-like receptor stimulation activates PKA to potentiate subthreshold L-type Ca^2+^ currents, yet it acts via PKC to suppress large amplitude Ca^2+^ spikes, thereby tuning Ca^2+^ currents to have the greatest activation in the voltage range necessary to produce spikes. Coupled with the D1 receptor–mediated increase in I_Nap_ and decrease in K^+^ currents, D1 receptor activation greatly prolongs the output of prefrontal pyramidal neurons (Neve et al., 2004; Beaulieu and Gainetdinov, 2011).

## Conclusion

A network pharmacology model–based bioinformatics algorithm was established to extract the core components group and decode the mechanisms of CHSGS in the treatment of depression. Compared with other published work, the effective intervention space construction strategy based on the novel node importance calculation method, CGFC prediction and validation strategy, and maximum targeting weight model for mechanism speculation were reported. Our research is a computational mining work based on pharmacological basic data, which provides a feasible scheme to reduce the verification scale for the experiment, provides methodological reference for the optimization of the core components group and interpretation of the molecular mechanism in the treatment of complex diseases using TCM.

However, there are some limitations in this study. Firstly, more components from the core group of functional components should be selected for validating the reliability of our approach. Secondly, the precise mechanisms were speculated by maximum targeting the weight model warrant further validation. Finally, the undirected network was utilized in our algorithm, which ignores the activation or inhibition effects of the targets.

In the era of big data and artificial intelligence, network pharmacology is helpful to study the TCM formula systematically. There are some suggestions for future research in network pharmacology. The dose-effect relationship of TCM components should be considered. Metabolites of TCM after entering the body may also be the material basis for exerting therapeutic effects, and the metabolic process of TCM in the body also needs to be considered in the network pharmacology study. In summary, there is still a long way to go in the quantification and digitization of TCM.

## Data Availability

The original contributions presented in the study are included in the article/Supplementary Material; further inquiries can be directed to the corresponding authors.

## Abbreviations

CCC, cumulative contribution coefficient; CHSGS, Chai-Hu-Shu-Gan-San; CGFC, core group of functional components; COVID-19, coronavirus disease-2019; C-T, component-target; CTPD, components-targets- pathogenic genes-disease; FBS, fetal bovine serum; GO, gene ontology; hERG, human Ether-à-go-go-Related Gene; KEGG, Kyoto Encyclopedia of Genes and Genomes; LDH, lactate dehydrogenase; MAOI, monoamine oxidase inhibitor; MTW, maximum targeting weight; NaSSA, noradrenergic and specific serotonergic antidepressants; OB, oral bioavailability; PLL, poly-L-polylysine; PPI, protein-protein interactions; SSRI, selective serotonin reuptake inhibitor; SNRI, serotonin norepinephrine reuptake inhibitor; TCM, traditional Chinese medicine; T-P, targets-pathogenic genes.

## References

[B1] Amberger J. S. Bocchini C. A. Schiettecatte F. Scott A. F. Hamosh A. (2015). OMIM.org: Online Mendelian Inheritance in Man (OMIM®), an Online Catalog of Human Genes and Genetic Disorders. Nucleic Acids Res. 43, D789–D798. 10.1093/nar/gku1205 25428349 PMC4383985

[B2] Assenov Y. Ramírez F. Schelhorn S. E. Lengauer T. Albrecht M. (2008). Computing Topological Parameters of Biological Networks. Bioinformatics 24 (2), 282–284. 10.1093/bioinformatics/btm554 18006545

[B3] Beaulieu J. M. Gainetdinov R. R. (2011). The Physiology, Signaling, and Pharmacology of Dopamine Receptors. Pharmacol. Rev. 63 (1), 182–217. 10.1124/pr.110.002642 21303898

[B4] Bondar J. Caye A. Chekroud A. M. Kieling C. (2020). Symptom Clusters in Adolescent Depression and Differential Response to Treatment: a Secondary Analysis of the Treatment for Adolescents with Depression Study Randomised Trial. Lancet Psychiatry 7 (4), 337–343. 10.1016/S2215-0366(20)30060-2 32199509

[B5] Chen C. Y. (2011). TCM Database@Taiwan: the World's Largest Traditional Chinese Medicine Database for Drug Screening In Silico. PLoS One 6, e15939. 10.1371/journal.pone.0015939 21253603 PMC3017089

[B6] Chen S. J. Cui M. C. (2017). Systematic Understanding of the Mechanism of Salvianolic Acid A via Computational Target Fishing. Molecules 22 (4), 644. 10.3390/molecules22040644 PMC6153743 28420179

[B7] Daina A. Michielin O. Zoete V. (2017). SwissADME: a Free Web Tool to Evaluate Pharmacokinetics, Drug-Likeness and Medicinal Chemistry Friendliness of Small Molecules. Sci. Rep. 7, 42717. 10.1038/srep42717 28256516 PMC5335600

[B8] Daina A. Michielin O. Zoete V. (2019). SwissTargetPrediction: Updated Data and New Features for Efficient Prediction of Protein Targets of Small Molecules. Nucleic Acids Res. 47, W357–W364. 10.1093/nar/gkz382 31106366 PMC6602486

[B9] Daina A. Zoete V. (2016). A BOILED-Egg to Predict Gastrointestinal Absorption and Brain Penetration of Small Molecules. ChemMedChem. 11 (11), 1117–1121. 10.1002/cmdc.201600182 27218427 PMC5089604

[B10] Delgado P. L. Moreno F. A. (2000). Role of Norepinephrine in Depression. J. Clin. Psychiatry 61 (Suppl. 1), 5–12. 10703757

[B11] Fimia G. M. Sassone-Corsi P. (2001). Cyclic AMP Signalling. J. Cel Sci. 114 (Pt 11), 1971–1972. 10.1242/jcs.114.11.1971 11493633

[B12] Gan Y. Zheng S. Zhao J. Zhang C. Gao T. Liao W. (2018). Protein Network Module-Based Identification of Key Pharmacological Pathways of Curcuma Phaeocaulis Val. Acting on Hepatitis. J. Ethnopharmacol 221, 10–19. 10.1016/j.jep.2018.03.004 29526702

[B13] Gao Y. Wang K. X. Wang P. Li X. Chen J. J. Zhou B. Y. (2020). A Novel Network Pharmacology Strategy to Decode Mechanism of Lang Chuang Wan in Treating Systemic Lupus Erythematosus. Front. Pharmacol. 11, 512877. 10.3389/fphar.2020.512877 33117150 PMC7562735

[B14] Gerlo S. Kooijman R. Beck I. M. Kolmus K. Spooren A. Haegeman G. (2011). Cyclic AMP: a Selective Modulator of NF-Κb Action. Cell Mol Life Sci. 68 (23), 3823–3841. 10.1007/s00018-011-0757-8 21744067 PMC11114830

[B15] Guo P. Cai C. Wu X. Fan X. Huang W. Zhou J. (2019b). An Insight into the Molecular Mechanism of Berberine towards Multiple Cancer Types through Systems Pharmacology. Front. Pharmacol. 10, 857. 10.3389/fphar.2019.00857 31447670 PMC6691338

[B16] Huang L. Xie D. Yu Y. Liu H. Shi Y. Shi T. (2018b). TCMID 2.0: a Comprehensive Resource for TCM. Nucleic Acids Res. 46 (D1), D1117–D1120. 10.1093/nar/gkx1028 29106634 PMC5753259

[B17] Huang X. Xu J. He J. Shi S. Yan H. Wang J. (2019). Pharmacokinetic Study of the Prokinetic ABCs Liquiritigenin, Naringenin and Hesperitin Following the Oral Administration of Si-Ni-San Decoction to Functional Dyspepsia Patients. Xenobiotica 49 (6), 708–717. 10.1080/00498254.2018.1493756 30286676

[B18] Kanehisa M. Goto S. (2000). KEGG: Kyoto Encyclopedia of Genes and Genomes. Nucleic Acids Res. 28 (1), 27–30. 10.1093/nar/28.1.27 10592173 PMC102409

[B19] Keiser M. J. Roth B. L. Armbruster B. N. Ernsberger P. Irwin J. J. Shoichet B. K. (2007). Relating Protein Pharmacology by Ligand Chemistry. Nat. Biotechnol. 25 (2), 197–206. 10.1038/nbt1284 17287757

[B20] Kerrien S. Aranda B. Breuza L. Bridge A. Broackes-Carter F. Chen C. (2012). The IntAct Molecular Interaction Database in 2012. Nucleic Acids Res. 40, D841–D846. 10.1093/nar/gkr1088 22121220 PMC3245075

[B21] Keshava Prasad T. S. Goel R. Kandasamy K. Keerthikumar S. Kumar S. Mathivanan S. (2009). Human Protein Reference Database--2009 updateHuman Protein Reference Database–2009 Update. Nucleic Acids Res. 37, D767–D772. 10.1093/nar/gkn892 18988627 PMC2686490

[B22] Kessler R. C. Berglund P. Demler O. Jin R. Merikangas K. R. Walters E. E. (2005). Lifetime Prevalence and Age-Of-Onset Distributions of DSM-IV Disorders in the National Comorbidity Survey Replication. Arch. Gen. Psychiatry 62 (6), 593–602. 10.1001/archpsyc.62.6.593 15939837

[B23] Kim H. J. Hwang I. K. Won M. H. (2007). Vanillin, 4-hydroxybenzyl Aldehyde and 4-hydroxybenzyl Alcohol Prevent Hippocampal CA1 Cell Death Following Global Ischemia. Brain Res. 1181, 130–141. 10.1016/j.brainres.2007.08.066 17945203

[B24] Kim S. K. Lee S. Lee M. K. Lee S. (2019). A Systems Pharmacology Approach to Investigate the Mechanism of Oryeong-San Formula for the Treatment of Hypertension. J. Ethnopharmacol 244, 112129. 10.1016/j.jep.2019.112129 31376514

[B25] Lee A. Y. Park W. Kang T. W. Cha M. H. Chun J. M. (2018). Network Pharmacology-Based Prediction of Active Compounds and Molecular Targets in Yijin-Tang Acting on Hyperlipidaemia and Atherosclerosis. J. Ethnopharmacol 221, 151–159. 10.1016/j.jep.2018.04.027 29698773

[B26] Lee S. K. Lee I. H. Kim H. J. Chang G. S. Chung J. E. No K. T. (2002). The PreADME Approach: Web-Based Program for Rapid Prediction of Physico-Chemical, Drug Absorption and Drug-like Properties. EuroQSAR 2002 Designing Drugs Crop Protectants: Process. Probl. solutions 3, 418–420.

[B27] Li B. Rui J. Ding X. Yang X. (2019). Exploring the Multicomponent Synergy Mechanism of Banxia Xiexin Decoction on Irritable Bowel Syndrome by a Systems Pharmacology Strategy. J. Ethnopharmacol 233, 158–168. 10.1016/j.jep.2018.12.033 30590198

[B28] Li S.-Q. Su Z.-H. Peng J.-B. Zou Z.-M. Yu C.-Y. (2010). *In Vitro* and *In Vivo* Antioxidant Effects and the Possible Relationship between the Antidepression Efficacy of Traditional Chinese Medicine Formulation Chaihu Shugan San. Chin. J. Nat. Medicines 8 (5), 353–361. 10.1016/s1875-5364(10)60042-8

[B29] Licata L. Briganti L. Peluso D. Perfetto L. Iannuccelli M. Galeota E. (2012). MINT, the Molecular Interaction Database: 2012 Update. Nucleic Acids Res. 40, D857–D861. 10.1093/nar/gkr930 22096227 PMC3244991

[B30] Liu H. Zeng L. Yang K. Zhang G. (2016). A Network Pharmacology Approach to Explore the Pharmacological Mechanism of Xiaoyao Powder on Anovulatory Infertility. Evid. Based Complement. Alternat Med. 2016, 2960372. 10.1155/2016/2960372 28074099 PMC5203871

[B31] Liu X. Vogt I. Haque T. Campillos M. (2013). HitPick: a Web Server for Hit Identification and Target Prediction of Chemical Screenings. Bioinformatics 29 (15), 1910–1912. 10.1093/bioinformatics/btt303 23716196

[B32] Lizcano J. M. Morrice N. Cohen P. (2000). Regulation of BAD by cAMP-dependent Protein Kinase Is Mediated via Phosphorylation of a Novel Site, Ser155. Biochem. J. 349 (Pt 2), 547–557. 10.1042/0264-6021:3490547 10880354 PMC1221178

[B33] Luo W. Brouwer C. (2013). Pathview: an R/Bioconductor Package for Pathway-Based Data Integration and Visualization. Bioinformatics 29 (14), 1830–1831. 10.1093/bioinformatics/btt285 23740750 PMC3702256

[B34] Luo Y. Li D. Liao Y. Cai C. Wu Q. Ke H. (2020). Systems Pharmacology Approach to Investigate the Mechanism of Kai-Xin-San in Alzheimer's Disease. Front. Pharmacol. 11, 381. 10.3389/fphar.2020.00381 32317964 PMC7147119

[B35] Malhi G. S. Mann J. J. (2018). Depression. Lancet 392 (10161), 2299–2312. 10.1016/S0140-6736(18)31948-2 30396512

[B36] Meng P. Han Y. Yang Q. Yang H. Zhu Q. Lin X. (2018). Xiaoyao Kangai Jieyu Fang, a Chinese Herbal Formulation, Ameliorates Cancer-Related Depression Concurrent with Breast Cancer in Mice via Promoting Hippocampal Synaptic Plasticity. Evid. Based Complement. Alternat Med. 2018, 3967642. 10.1155/2018/3967642 30581482 PMC6276466

[B37] Mustafa T. (2013). Pituitary Adenylate Cyclase-Activating Polypeptide (PACAP): a Master Regulator in central and Peripheral Stress Responses. Adv. Pharmacol. 68, 445–457. 10.1016/B978-0-12-411512-5.00021-X 24054157

[B38] Narasingam M. Vijeepallam K. Mohamed Z. Pandy V. (2017). Anxiolytic- and Antidepressant-like Activities of a Methanolic Extract of *Morinda citrifolia* Linn. (Noni) Fruit in Mice: Involvement of Benzodiazepine-GABAAergic, Serotonergic and Adrenergic Systems. Biomed. Pharmacother. 96, 944–952. 10.1016/j.biopha.2017.11.148 29217165

[B39] Neve K. A. Seamans J. K. Trantham-Davidson H. (2004). Dopamine Receptor Signaling. J. Recept Signal. Transduct Res. 24, 165–205. 10.1081/rrs-200029981 15521361

[B40] O'Boyle N. M. Banck M. James C. A. Morley C. Vandermeersch T. Hutchison G. R. (2011). Open Babel: An Open Chemical Toolbox. J. Cheminform 3, 33. 10.1186/1758-2946-3-33 21982300 PMC3198950

[B41] Oughtred R. Stark C. Breitkreutz B. J. Rust J. Boucher L. Chang C. (2019). The BioGRID Interaction Database: 2019 Update. Nucleic Acids Res. 47 (D1), D529–D541. 10.1093/nar/gky1079 30476227 PMC6324058

[B42] Piñero J. Bravo À. Queralt-Rosinach N. Gutiérrez-Sacristán A. Deu-Pons J. Centeno E. (2017). DisGeNET: a Comprehensive Platform Integrating Information on Human Disease-Associated Genes and Variants. Nucleic Acids Res. 45 (D1), D833–D839. 10.1093/nar/gkw943 27924018 PMC5210640

[B43] Ren Q. Li M. Deng Y. Lu A. Lu J. (2021). Triptolide Delivery: Nanotechnology-Based Carrier Systems to Enhance Efficacy and Limit Toxicity. Pharmacol. Res. 165, 105377. 10.1016/j.phrs.2020.105377 33484817

[B44] Ru J. Li P. Wang J. Zhou W. Li B. Huang C. (2014). TCMSP: a Database of Systems Pharmacology for Drug Discovery from Herbal Medicines. J. Cheminform 6, 13. 10.1186/1758-2946-6-13 24735618 PMC4001360

[B45] Safran M. Dalah I. Alexander J. Rosen N. Iny Stein T. Shmoish M. (2010). GeneCards Version 3: the Human Gene Integrator. Database (Oxford) 2010, baq020. 10.1093/database/baq020 20689021 PMC2938269

[B46] Salwinski L. Miller C. S. Smith A. J. Pettit F. K. Bowie J. U. Eisenberg D. (2004). The Database of Interacting Proteins: 2004 Update. Nucleic Acids Res. 32, D449–D451. 10.1093/nar/gkh086 14681454 PMC308820

[B47] Shannon P. Markiel A. Ozier O. Baliga N. S. Wang J. T. Ramage D. (2003). Cytoscape: a Software Environment for Integrated Models of Biomolecular Interaction Networks. Genome Res. 13 (11), 2498–2504. 10.1101/gr.1239303 14597658 PMC403769

[B48] Shi B. Luo J. Fang Y. Liu X. Rao Z. Liu R. (2019). Xiaoyao Pills Prevent Lipopolysaccharide-Induced Depression by Inhibiting Inflammation and Protecting Nerves. Front. Pharmacol. 10, 1324. 10.3389/fphar.2019.01324 31798446 PMC6863983

[B51] Su Z. H. Li S. Q. Zou G. A. Yu C. Y. Sun Y. G. Zhang H. W. (2011). Urinary Metabonomics Study of Anti-depressive Effect of Chaihu-Shu-Gan-San on an Experimental Model of Depression Induced by Chronic Variable Stress in Rats. J. Pharm. Biomed. Anal. 55 (3), 533–539. 10.1016/j.jpba.2011.02.013 21398066

[B53] Szklarczyk D. Gable A. L. Lyon D. Junge A. Wyder S. Huerta-Cepas J. (2019). STRING V11: Protein-Protein Association Networks with Increased Coverage, Supporting Functional Discovery in Genome-wide Experimental Datasets. Nucleic Acids Res. 47 (D1), D607–D613. 10.1093/nar/gky1131 30476243 PMC6323986

[B54] Virtanen S. Kuja-Halkola R. Mataix-Cols D. Jayaram-Lindstrom N. D'Onofrio B. M. Larsson H. (2019). Comorbidity of Substance Misuse with Anxiety-Related and Depressive Disorders: a Genetically Informative Population Study of 3 Million Individuals in Sweden. Psychol. Med. 50 (10), 1706–1715. 10.1017/s0033291719001788 31328718

[B55] Voglis G. Tavernarakis N. (2006). The Role of Synaptic Ion Channels in Synaptic Plasticity. EMBO Rep. 7 (11), 1104–1110. 10.1038/sj.embor.7400830 17077866 PMC1679792

[B56] Wang C. Ren Q. Chen X. T. Song Z. Q. Ning Z. C. Gan J. H. (2018). System Pharmacology-Based Strategy to Decode the Synergistic Mechanism of Zhi-Zhu Wan for Functional Dyspepsia. Front. Pharmacol. 9, 841. 10.3389/fphar.2018.00841 30127739 PMC6087764

[B57] Wang K. X. Chen Y. P. Lu A. P. Du G. H. Qin X. M. Guan D. G. (2021). A Metabolic Data-Driven Systems Pharmacology Strategy for Decoding and Validating the Mechanism of Compound Kushen Injection against HCC. J. Ethnopharmacol 274, 114043. 10.1016/j.jep.2021.114043 33753143

[B58] Wang K. X. Gao Y. Gong W. X. Ye X. F. Fan L. Y. Wang C. (2020b). A Novel Strategy for Decoding and Validating the Combination Principles of Huanglian Jiedu Decoction from Multi-Scale Perspective. Front. Pharmacol. 11, 567088. 10.3389/fphar.2020.567088 33424585 PMC7789881

[B59] Wang K. X. Gao Y. Lu C. Li Y. Zhou B. Y. Qin X. M. (2020a). Uncovering the Complexity Mechanism of Different Formulas Treatment for Rheumatoid Arthritis Based on a Novel Network Pharmacology Model. Front. Pharmacol. 11, 1035. 10.3389/fphar.2020.01035 32754034 PMC7365894

[B60] Wang M. Bi Y. Zeng S. Liu Y. Shao M. Liu K. (2019a). Modified Xiaoyao San Ameliorates Depressive-like Behaviors by Triggering Autophagosome Formation to Alleviate Neuronal Apoptosis. Biomed. Pharmacother. 111, 1057–1065. 10.1016/j.biopha.2018.12.141 30841419

[B61] Wang S. Hu S. Zhang C. Qiu J. Li Y. (2011). Effect of Chaihu Shugan San and its Components on Expression of ERK1/2 mRNA in the hippocampus of Rats with Chronic Mild Unpredicted Stress Depression, Zhong Nan da Xue Xue Bao. Yi Xue Ban, 36. 93–100. 10.3969/j.issn.1672-7347.2011.02.001 21368416

[B62] Wang Y. M. Xu H. Xu J. Y. Li G. W. Xu S. C. (2013). Effect of Vanillin Sinff on Relieving Depression-Likebehaviors in Mice and Discussion on the Possible Mechanism. Anat. Clin. 18 (5), 394–398.

[B63] Wu Y. Zhang F. Yang K. Fang S. Bu D. Li H. (2019). SymMap: an Integrative Database of Traditional Chinese Medicine Enhanced by Symptom Mapping. Nucleic Acids Res. 47 (D1), D1110–D1117. 10.1093/nar/gky1021 30380087 PMC6323958

[B64] Xu H. Y. Zhang Y. Q. Liu Z. M. Chen T. Lv C. Y. Tang S. H. (2019). ETCM: an Encyclopaedia of Traditional Chinese Medicine. Nucleic Acids Res. 47 (D1), D976–D982. 10.1093/nar/gky987 30365030 PMC6323948

[B65] Yang L. Fan L. Wang K. Chen Y. Liang L. Qin X. (2021). Analysis of Molecular Mechanism of Erxian Decoction in Treating Osteoporosis Based on Formula Optimization Model. Oxid Med. Cel Longev. 2021, 6641838. 10.1155/2021/6641838 PMC8238601 34239693

[B66] Yu G. Wang L. G. Han Y. He Q. Y. (2012). clusterProfiler: an R Package for Comparing Biological Themes Among Gene Clusters. OMICS 16 (5), 284–287. 10.1089/omi.2011.0118 22455463 PMC3339379

[B67] Zong Y. Chen T. Dong H. Zhu L. Ju W. (2019). Si-Ni-San Prevents Reserpine-Induced Depression by Inhibiting Inflammation and Regulating CYP450 Enzymatic Activity. Front. Pharmacol. 10, 1518. 10.3389/fphar.2019.01518 32009949 PMC6978689

