# Supplementary figures and images for: Computational Network Pharmacology–Based Strategy to Capture Key Functional Components and Decode the Mechanism of Chai-Hu-Shu-Gan-San in Treating Depression
Source: Front Pharmacol. 2021 Nov 12;12:782060. doi: 10.3389/fphar.2021.782060 (PMC8633106; doi:10.3389/fphar.2021.782060)

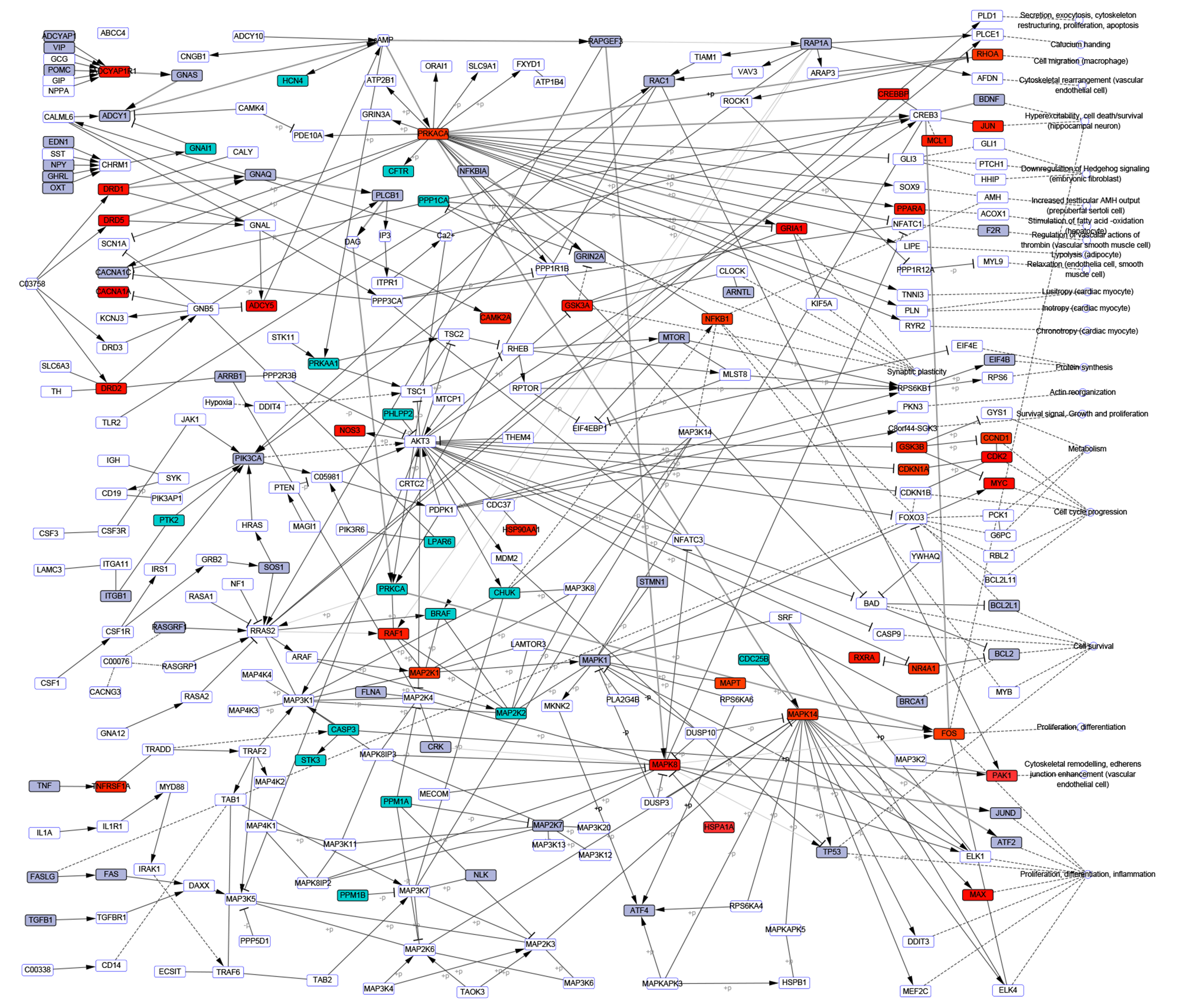

Supplement: Supplementary file 2 [file Image1.TIF]
