# Supplementary material for: Computational Network Pharmacology–Based Strategy to Capture Key Functional Components and Decode the Mechanism of Chai-Hu-Shu-Gan-San in Treating Depression
Source: Front Pharmacol. 2021 Nov 12;12:782060. doi: 10.3389/fphar.2021.782060 (PMC8633106; doi:10.3389/fphar.2021.782060)
Supplement: Supplementary file 3 [file Table2.DOCX]

Table S2 The information on chemical analysis of the herbs from the literature in CHSGS.

| Formula/Herbs | Method | Component | Concentration | Ref. |
| --- | --- | --- | --- | --- |
| Chaihu Shugan Powder | HPLC/DAD | saikosaponin A | 0.7626 mg/g | Wang et al., 2010 |
|  |  | paeoniflorin | 1.189 mg/g |  |
|  |  | hesperidin | 4.737 mg/g |  |
|  |  | ferulic acid | 0.1826 mg/g |  |
| *Citrus Reticulata* (Chenpi) | RP-HPLC | hesperidin | 50.5865 mg/g | Lin et al., 2010 |
|  |  | nobiletin | 6.6762 mg/g |  |
|  |  | tangeretin | 4.5343 mg/g |  |
| *Radix Bupleuri* (Chaihu) | HPLC-ELSD | saikosaponins a | 3.1875 mg/g | Zhang et al., 2007 |
|  |  | saikosaponins c | 1.0133 mg/g |  |
|  |  | saikosaponins d | 1.5875 mg/g |  |
|  |  | saikosaponins f | 0.5667 mg/g |  |
| *Chuanxiong Rhizoma* (Chuanxiong) | HPLC | ferulic acid | 2.94 mg/g | Liu et al., 2014 |
|  |  | senkyunolide I | 0.70 mg/g |  |
|  |  | senkyunolide H | 2.19 mg/g |  |
|  |  | senkyunolide A | 15.21 mg/g |  |
|  |  | ligustilide | 30.25 mg/g |  |
| *Cyperi Rhizoma* (Xiangfu) | HPLC | nootkatone | 0.1535 mg/g | Ji et al., 2015 |
|  |  | cyperotundone | 2.7228 mg/g |  |
|  |  | α-cyperone | 1.4049 mg/g |  |
| *Aurantii Fructus* (Zhike) | HPLC | hesperidin | 49.2533 mg/g | Li et al., 2002 |
|  |  | naringin | 6.3233 mg/g |  |
| *Paeoniae Radix Alba* (Baishao) | HPLC | Gallic acid | 2.33 mg/g | Li et al., 2011 |
|  |  | Hydroxyl-paeoniflorin | 1.89 mg/g |  |
|  |  | Catechin | 0.03 mg/g |  |
|  |  | Albiflorin | 4.44 mg/g |  |
|  |  | Paeoniflorin | 4.81 mg/g |  |
|  |  | Benzoic acid | 0.03 mg/g |  |
|  |  | 1, 2, 3, 4, 6 -pentagalloylglucose | 4.80 mg/g |  |
|  |  | Benzoyl -paeoniflorin | 0.11 mg/g |  |
|  |  | Paeonol | 0.07 mg/g |  |
| *Glycyrrhiza uralensis* (Gancao) | HPLC | Glycyrrhizin | 97.49 mg/g | Chen et al., 2009 |
|  |  | Liquiritin | 102.83 mg/g |  |
|  |  | Lsoliquritigenin | 98.30 mg/g |  |
