# Supplementary material for: Computational Network Pharmacology–Based Strategy to Capture Key Functional Components and Decode the Mechanism of Chai-Hu-Shu-Gan-San in Treating Depression
Source: Front Pharmacol. 2021 Nov 12;12:782060. doi: 10.3389/fphar.2021.782060 (PMC8633106; doi:10.3389/fphar.2021.782060)
Supplement: Supplementary file 5 [file Table4.DOCX]

Table S4. Active components in CHSGS for further analysis after ADME screening.

| **ID** | **molecule_name** | **Hdon** | **Hacc** | **OB (%)** | **RBN** | **logP** | **MW** | **GI absorption** | **hERG_inhibition** | **Carcino_Mouse** |
| --- | --- | --- | --- | --- | --- | --- | --- | --- | --- | --- |
| CHSGS1 | (+/-)-Isoborneol | 1 | 1 | 86.98 | 0 | 2.83 | 154.25 | High | low_risk | negative |
| CHSGS2 | quercetin | 5 | 7 | 46.43 | 1 | 1.07 | 302.24 | High | medium_risk | negative |
| CHSGS3 | PHB | 2 | 3 | 30.15 | 1 | 1.58 | 138.12 | High | low_risk | negative |
| CHSGS4 | vanillic acid | 2 | 4 | 35.47 | 2 | 1.7 | 168.15 | High | low_risk | negative |
| CHSGS5 | Nonanal | 0 | 1 | 40.28 | 7 | 3.81 | 142.24 | High | low_risk | negative |
| CHSGS6 | (L)-alpha-Terpineol | 1 | 1 | 48.8 | 1 | 3.17 | 154.25 | High | low_risk | negative |
| CHSGS7 | CAM | 0 | 1 | 67.17 | 0 | 2.85 | 152.23 | High | low_risk | negative |
| CHSGS8 | 2-[(2S,5S,6S)-6,10-dimethylspiro[4.5]dec-9-en-2-yl]propan-2-ol | 1 | 1 | 37.62 | 1 | 4.84 | 222.37 | High | low_risk | negative |
| CHSGS9 | L-Bornyl acetate | 0 | 2 | 65.52 | 2 | 3.5 | 196.29 | High | low_risk | negative |
| CHSGS10 | (R)-linalool | 1 | 1 | 39.8 | 4 | 2.68 | 154.25 | High | low_risk | negative |
| CHSGS11 | BOX | 0 | 2 | 31.55 | 1 | 1.72 | 122.12 | High | medium_risk | negative |
| CHSGS12 | delta-Terpineol | 1 | 1 | 55.11 | 1 | 2.75 | 154.25 | High | low_risk | negative |
| CHSGS13 | Jaranol | 2 | 6 | 50.83 | 3 | 2.8 | 314.29 | High | medium_risk | negative |
| CHSGS14 | ()-Borneol | 1 | 1 | 81.8 | 0 | 2.83 | 154.25 | High | low_risk | negative |
| CHSGS15 | o-Thymol | 1 | 1 | 43.28 | 1 | 3.2 | 150.22 | High | low_risk | negative |
| CHSGS16 | l-carvone | 0 | 1 | 49.47 | 1 | 2.77 | 150.22 | High | low_risk | negative |
| CHSGS17 | isorhamnetin | 4 | 7 | 49.6 | 2 | 1.31 | 316.26 | High | medium_risk | negative |
| CHSGS18 | formononetin | 1 | 4 | 69.67 | 2 | 3.01 | 268.26 | High | medium_risk | negative |
| CHSGS19 | Calycosin | 2 | 5 | 47.75 | 2 | 2.82 | 284.26 | High | medium_risk | negative |
| CHSGS20 | kaempferol | 4 | 6 | 41.88 | 1 | 1.23 | 286.24 | High | medium_risk | negative |
| CHSGS21 | CHEBI:39932 | 1 | 1 | 32.79 | 5 | 2.43 | 128.21 | High | low_risk | negative |
| CHSGS22 | (+)-catechin | 5 | 6 | 54.83 | 1 | 1.02 | 290.27 | High | medium_risk | negative |
| CHSGS23 | Vestitol | 2 | 4 | 74.66 | 2 | 2.89 | 272.3 | High | medium_risk | negative |
| CHSGS24 | 3,4,5-trihydroxybenzoic acid | 4 | 5 | 31.69 | 1 | 1.17 | 170.12 | High | low_risk | negative |
| CHSGS25 | (1R,5S)-7,7-dimethyl-4-bicyclo[3.1.1]hept-3-enecarboxaldehyde | 0 | 1 | 40.64 | 1 | 3.03 | 150.22 | High | low_risk | negative |
| CHSGS26 | vanillin | 1 | 3 | 52 | 2 | 1.31 | 152.15 | High | low_risk | negative |
| CHSGS27 | hexanal | 0 | 1 | 55.71 | 4 | 2.37 | 100.16 | High | low_risk | negative |
| CHSGS28 | ()-Menthol | 1 | 1 | 59.33 | 1 | 2.68 | 156.27 | High | low_risk | negative |
| CHSGS29 | patchouli alcohol | 1 | 1 | 101.96 | 0 | 4.19 | 222.37 | High | low_risk | negative |
| CHSGS30 | β-terpineol | 1 | 1 | 47.89 | 1 | 2.73 | 154.25 | High | low_risk | negative |
| CHSGS31 | 2-heptanone | 0 | 1 | 46.56 | 4 | 1.92 | 114.19 | High | low_risk | negative |
| CHSGS32 | WLN: VHR | 0 | 1 | 32.63 | 1 | 1.6 | 106.12 | High | medium_risk | negative |
| CHSGS33 | (S)-Matsutake alcohol | 1 | 1 | 40.11 | 5 | 2.43 | 128.21 | High | low_risk | negative |
| CHSGS34 | Hyacinthin | 0 | 1 | 38.65 | 2 | 1.75 | 120.15 | High | medium_risk | negative |
| CHSGS35 | l-Menthone | 0 | 1 | 57.9 | 1 | 2.65 | 154.25 | High | low_risk | negative |
| CHSGS36 | HMF | 1 | 3 | 45.07 | 2 | -0.17 | 126.11 | High | low_risk | negative |
| CHSGS37 | CYH | 0 | 1 | 74.99 | 0 | 1.03 | 98.14 | High | low_risk | negative |
| CHSGS38 | isocurcumenol | 1 | 2 | 97.67 | 0 | 2.37 | 234.33 | High | low_risk | negative |
| CHSGS39 | cuminal | 0 | 1 | 38.29 | 2 | 2.73 | 148.2 | High | medium_risk | negative |
| CHSGS40 | ()-Bornyl acetate | 0 | 2 | 65.55 | 2 | 3.5 | 196.29 | High | low_risk | negative |
| CHSGS41 | (S)-2,2,3-Trimethylcyclopent-3-ene-1-acetaldehyde | 0 | 1 | 45.18 | 2 | 3.17 | 152.23 | High | low_risk | negative |
| CHSGS42 | trans-pinocamphone (pinocamphone) | 0 | 1 | 50.35 | 0 | 2.25 | 152.23 | High | low_risk | negative |
| CHSGS43 | (1S,5R)-7,7-dimethyl-4-bicyclo[3.1.1]hept-3-enecarboxaldehyde | 0 | 1 | 41.25 | 1 | 3.03 | 150.22 | High | low_risk | negative |
| CHSGS44 | (1R)-()-Nopinone | 0 | 1 | 57.86 | 0 | 2.06 | 138.21 | High | low_risk | negative |
| CHSGS45 | 80-57-9 | 0 | 1 | 50.63 | 0 | 2.3 | 150.22 | High | low_risk | negative |
| CHSGS46 | WLN: Q1R | 1 | 1 | 58.68 | 1 | 1.07 | 108.14 | High | medium_risk | negative |
| CHSGS47 | 49070_FLUKA | 1 | 1 | 85.51 | 0 | 3.15 | 222.37 | High | low_risk | negative |
| CHSGS48 | Inermine | 1 | 5 | 75.18 | 0 | 2.19 | 284.26 | High | medium_risk | negative |
| CHSGS49 | Hypnon | 0 | 1 | 48.19 | 1 | 1.65 | 120.15 | High | medium_risk | negative |
| CHSGS50 | α-cubebol | 1 | 1 | 64.81 | 0 | 3.52 | 208.34 | High | low_risk | negative |
| CHSGS51 | IFP | 3 | 3 | 72.87 | 2 | -1.93 | 92.09 | High | low_risk | negative |
| CHSGS52 | ICO | 2 | 2 | 33.86 | 1 | 1.79 | 161.16 | High | medium_risk | negative |
| CHSGS53 | EUG | 1 | 2 | 38.39 | 2 | 1.84 | 150.17 | High | medium_risk | negative |
| CHSGS54 | adenine | 3 | 4 | 62.81 | 0 | -0.38 | 135.13 | High | medium_risk | negative |
| CHSGS55 | DFV | 2 | 4 | 32.76 | 1 | 2.79 | 256.25 | High | medium_risk | negative |
| CHSGS56 | salicylic acid | 2 | 3 | 32.13 | 1 | 1.96 | 138.12 | High | low_risk | negative |
| CHSGS57 | Izoforon | 0 | 1 | 44.98 | 0 | 1.9 | 138.21 | High | low_risk | negative |
| CHSGS58 | 2,2-dimethylcyclohexanol | 1 | 1 | 82.54 | 0 | 2.23 | 128.21 | High | low_risk | negative |
| CHSGS59 | BU3 | 2 | 2 | 34.87 | 1 | -0.59 | 90.12 | High | low_risk | negative |
| CHSGS60 | Dibutylphenol | 1 | 1 | 38.9 | 2 | 4.9 | 206.32 | High | low_risk | negative |
| CHSGS61 | ()-trans-Myrtanol | 1 | 1 | 49.66 | 1 | 2.53 | 154.25 | High | low_risk | negative |
| CHSGS62 | Acetyl oxide | 0 | 3 | 45.13 | 2 | 0.11 | 102.09 | High | low_risk | negative |
| CHSGS63 | Methylgallate | 3 | 5 | 30.91 | 2 | 1.01 | 184.15 | High | low_risk | negative |
| CHSGS64 | (3S,3aR,5S,6S,7aR)-5,6-dihydroxy-3,6-dimethyl-3,3a,4,5,7,7a-hexahydrobenzofuran-2-one | 2 | 4 | 96.64 | 0 | 0.07 | 200.23 | High | low_risk | negative |
| CHSGS65 | paeoniflorgenone | 1 | 6 | 87.59 | 4 | 0.86 | 318.32 | High | medium_risk | negative |
| CHSGS66 | paeoniflorin_qt | 2 | 6 | 68.18 | 4 | 0.69 | 318.32 | High | medium_risk | negative |
| CHSGS67 | albiflorin_qt | 2 | 6 | 66.64 | 4 | 0.53 | 318.32 | High | medium_risk | negative |
| CHSGS68 | (3R,3aR,6S,7aR)-6-hydroxy-3,6-dimethyl-3a,4,7,7a-tetrahydro-3H-benzofuran-2,5-dione | 1 | 4 | 104.94 | 0 | 0.02 | 198.22 | High | low_risk | negative |
| CHSGS69 | (3aR,6S,7aR)-6-hydroxy-6-methyl-3-methylene-3a,4,7,7a-tetrahydrobenzofuran-2,5-dione | 1 | 4 | 97.79 | 0 | -0.42 | 196.2 | High | low_risk | negative |
| CHSGS70 | 24253-30-3 | 0 | 1 | 74.2 | 3 | 1.21 | 98.14 | High | low_risk | negative |
| CHSGS71 | Alloisoimperatorin | 1 | 4 | 34.8 | 2 | 3.48 | 270.28 | High | medium_risk | negative |
| CHSGS72 | Ammidin | 0 | 4 | 34.55 | 3 | 3.78 | 270.28 | High | medium_risk | negative |
| CHSGS73 | isoimperatorin | 0 | 4 | 45.46 | 3 | 3.7 | 270.28 | High | medium_risk | negative |
| CHSGS74 | Marmesin | 1 | 4 | 50.28 | 1 | 2.1 | 246.26 | High | low_risk | negative |
| CHSGS75 | Pulegone | 0 | 1 | 51.6 | 0 | 2.36 | 152.23 | High | low_risk | negative |
| CHSGS76 | scoparone | 0 | 4 | 74.75 | 2 | 1.91 | 206.19 | High | low_risk | negative |
| CHSGS77 | thymol | 1 | 1 | 41.47 | 1 | 3.16 | 150.22 | High | low_risk | negative |
| CHSGS78 | hexanoic acid | 1 | 2 | 73.08 | 4 | 1.88 | 116.16 | High | low_risk | negative |
| CHSGS79 | Isovanillic acid | 2 | 4 | 39.42 | 2 | 1.81 | 168.15 | High | low_risk | negative |
| CHSGS80 | Senkyunolide-N | 2 | 4 | 37.27 | 3 | 0.94 | 226.27 | High | low_risk | negative |
| CHSGS81 | Valerophenone | 0 | 1 | 42.58 | 4 | 2.94 | 162.23 | High | medium_risk | negative |
| CHSGS82 | Isobutyrophenone | 0 | 1 | 80.37 | 2 | 2.54 | 148.2 | High | medium_risk | negative |
| CHSGS83 | Myricanone | 2 | 5 | 40.6 | 2 | 3.95 | 356.41 | High | low_risk | negative |
| CHSGS84 | p-Cymen-8-ol | 1 | 1 | 32.26 | 1 | 2.53 | 150.22 | High | low_risk | negative |
| CHSGS85 | Perlolyrine | 2 | 3 | 65.95 | 2 | 2.66 | 264.28 | High | medium_risk | negative |
| CHSGS86 | senkyunolide-C | 1 | 3 | 46.8 | 2 | 3.49 | 204.22 | High | low_risk | negative |
| CHSGS87 | senkyunolide-E | 1 | 3 | 34.4 | 2 | 1.93 | 204.22 | High | medium_risk | negative |
| CHSGS88 | senkyunolide-F | 1 | 3 | 40.35 | 2 | 2.16 | 206.24 | High | low_risk | negative |
| CHSGS89 | 1-Acetyl-beta-carboline | 1 | 2 | 67.12 | 1 | 2.53 | 210.23 | High | medium_risk | negative |
| CHSGS90 | sinapic acid | 2 | 5 | 64.15 | 4 | 1.63 | 224.21 | High | low_risk | negative |
| CHSGS91 | wallichilide | 0 | 5 | 42.31 | 8 | 4.8 | 412.52 | High | low_risk | negative |
| CHSGS92 | 1-terpineol | 1 | 1 | 49.83 | 1 | 2.91 | 154.25 | High | low_risk | negative |
| CHSGS93 | WLN: 2VR | 0 | 1 | 60.17 | 2 | 2.15 | 134.18 | High | medium_risk | negative |
| CHSGS94 | 2,2,3-Trimethylcyclopent-3-ene-1-carboxaldehyde | 0 | 1 | 42.64 | 1 | 2.75 | 138.21 | High | low_risk | negative |
| CHSGS95 | 3,4-epoxy-2,2,7,7-tetramethyl-octane | 0 | 1 | 66.87 | 4 | 4.06 | 184.32 | High | medium_risk | negative |
| CHSGS96 | 3-cyclohexen-1-ol | 1 | 1 | 70.57 | 0 | 0.96 | 98.14 | High | low_risk | negative |
| CHSGS97 | 4,7-Dihydroxy-3-butylphthalide | 2 | 4 | 106.09 | 3 | 2.69 | 222.24 | High | low_risk | negative |
| CHSGS98 | Aromadendrene oxide 2 | 0 | 1 | 65.1 | 0 | 2.68 | 220.35 | High | medium_risk | negative |
| CHSGS99 | L-valyl-L-valinc-achydride | 4 | 4 | 40.18 | 6 | 0 | 214.3 | High | low_risk | negative |
| CHSGS100 | Glycyrol | 2 | 6 | 90.78 | 3 | 4.06 | 366.36 | High | medium_risk | negative |
| CHSGS101 | Hesperetin | 3 | 6 | 70.31 | 2 | 2.52 | 302.28 | High | medium_risk | negative |
| CHSGS102 | (s)-carvone | 0 | 1 | 47.43 | 1 | 2.77 | 150.22 | High | low_risk | negative |
| CHSGS103 | (-)-isomenthone | 0 | 1 | 61.19 | 1 | 2.65 | 154.25 | High | low_risk | negative |
| CHSGS104 | PTL | 0 | 1 | 59.53 | 3 | 1.41 | 86.13 | High | low_risk | negative |
| CHSGS105 | Isovaleral | 0 | 1 | 44.71 | 2 | 1.29 | 86.13 | High | low_risk | negative |
| CHSGS106 | Medicarpin | 1 | 4 | 49.22 | 1 | 3.07 | 270.28 | High | medium_risk | negative |
| CHSGS107 | Phellopterin | 0 | 5 | 40.19 | 4 | 3.49 | 300.31 | High | medium_risk | negative |
| CHSGS108 | Hexenal | 0 | 1 | 46.01 | 3 | 1.8 | 98.14 | High | low_risk | negative |
| CHSGS109 | Apocynin | 1 | 3 | 31.71 | 2 | 1.62 | 166.17 | High | low_risk | negative |
| CHSGS110 | Pinocembrin | 2 | 4 | 64.72 | 1 | 2.85 | 256.25 | High | medium_risk | negative |
| CHSGS111 | (1S,3R,5S)-6,6-dimethyl-2-methylene-3-norpinanol | 1 | 1 | 53.99 | 0 | 2.24 | 152.23 | High | low_risk | negative |
| CHSGS112 | WLN: QV4 | 1 | 2 | 70.74 | 3 | 1.34 | 102.13 | High | low_risk | negative |
| CHSGS113 | IPH | 1 | 1 | 36.05 | 0 | 1.39 | 94.11 | High | medium_risk | negative |
| CHSGS114 | Chryseriol | 3 | 6 | 35.85 | 2 | 2.53 | 300.26 | High | medium_risk | negative |
| CHSGS115 | ZINC02571348 | 1 | 1 | 39.15 | 6 | 2.93 | 142.24 | High | low_risk | negative |
| CHSGS116 | m-Methylacetophenone | 0 | 1 | 40.63 | 1 | 2.08 | 134.18 | High | low_risk | negative |
| CHSGS117 | Prangenidin | 1 | 4 | 36.31 | 2 | 3.56 | 270.28 | High | medium_risk | negative |
| CHSGS118 | Lupiwighteone | 3 | 5 | 51.64 | 3 | 3.23 | 338.35 | High | medium_risk | negative |
| CHSGS119 | 7-Methoxy-2-methyl isoflavone | 0 | 3 | 42.56 | 2 | 3.48 | 266.29 | High | medium_risk | negative |
| CHSGS120 | 2-Caren-10-al | 0 | 1 | 44.74 | 1 | 2.16 | 150.22 | High | low_risk | negative |
| CHSGS121 | Visnagin | 0 | 4 | 44.25 | 1 | 1.92 | 230.22 | High | low_risk | negative |
| CHSGS122 | (4aR,7R)-7-isopropenyl-1,4a-dimethyl-3,4,5,6,7,8-hexahydronaphthalen-2-one | 0 | 1 | 35.37 | 1 | 3.92 | 218.33 | High | low_risk | negative |
| CHSGS123 | 4,7-dimethyl-1-tetralone | 0 | 1 | 34.41 | 0 | 2.88 | 174.24 | High | low_risk | negative |
| CHSGS124 | Cyperolone | 1 | 2 | 42.59 | 2 | 2.76 | 236.35 | High | low_risk | negative |
| CHSGS125 | cyperotundone | 0 | 1 | 55.6 | 0 | 3.93 | 218.33 | High | low_risk | negative |
| CHSGS126 | Dimethyl tetrasulfide | 0 | 0 | 51.34 | 3 | 1.04 | 158.33 | High | medium_risk | negative |
| CHSGS127 | (1S,3aS,4S,7R,8aS)-7-isopropenyl-1,4-dimethyl-2,3,3a,5,6,7,8,8a-octahydroazulene-1,4-diol | 2 | 2 | 72 | 1 | 2.84 | 238.37 | High | low_risk | negative |
| CHSGS128 | Isodalbergin | 1 | 4 | 35.45 | 2 | 3.76 | 268.26 | High | medium_risk | negative |
| CHSGS129 | isokobusone | 1 | 2 | 39.63 | 0 | 1.93 | 222.32 | High | low_risk | negative |
| CHSGS130 | Khell | 0 | 5 | 33.19 | 2 | 1.78 | 260.24 | High | low_risk | negative |
| CHSGS131 | Khellol | 1 | 5 | 68.36 | 2 | 0.88 | 246.22 | High | low_risk | negative |
| CHSGS132 | ()-Nootkatone | 0 | 1 | 33.04 | 1 | 3.89 | 218.33 | High | low_risk | negative |
| CHSGS133 | rotundenol | 1 | 1 | 74.95 | 0 | 3.67 | 220.35 | High | low_risk | negative |
| CHSGS134 | Hyndarin | 0 | 5 | 73.94 | 4 | 3.09 | 355.43 | High | low_risk | negative |
| CHSGS135 | sugebiol | 2 | 2 | 73.37 | 0 | 2.22 | 236.35 | High | low_risk | negative |
| CHSGS136 | sugetriol | 3 | 3 | 68.87 | 0 | 0.79 | 252.35 | High | low_risk | negative |
| CHSGS137 | naringenin | 3 | 5 | 59.29 | 1 | 2.47 | 272.25 | High | medium_risk | negative |
| CHSGS138 | acetic acid | 1 | 2 | 47.87 | 0 | -0.12 | 60.05 | High | low_risk | negative |
| CHSGS139 | Heptenoic acid | 1 | 2 | 36.1 | 4 | 2.41 | 128.17 | High | low_risk | negative |
| CHSGS140 | (2R)-2-methylcyclopentan-1-one | 0 | 1 | 60.04 | 0 | 0.98 | 98.14 | High | low_risk | negative |
| CHSGS141 | Nonenoic acid | 1 | 2 | 65.17 | 6 | 3.53 | 156.22 | High | low_risk | negative |
| CHSGS142 | 2-Octenic acid | 1 | 2 | 43.49 | 5 | 3.01 | 142.2 | High | low_risk | negative |
| CHSGS143 | 3,5,6,7-tetramethoxy-2-(3,4,5-trimethoxyphenyl)chromone | 0 | 9 | 31.97 | 8 | 2.59 | 432.42 | High | low_risk | negative |
| CHSGS144 | Heptan-3-on | 0 | 1 | 68.44 | 4 | 2.09 | 114.19 | High | low_risk | negative |
| CHSGS145 | Ayapanin | 0 | 3 | 41.55 | 1 | 2.06 | 176.17 | High | medium_risk | negative |
| CHSGS146 | 8-NONENOIC ACID | 1 | 2 | 52.31 | 7 | 2.84 | 156.22 | High | low_risk | negative |
| CHSGS147 | Longikaurin A | 3 | 5 | 47.72 | 0 | 1.04 | 348.43 | High | low_risk | negative |
| CHSGS148 | Octalupine | 1 | 4 | 47.82 | 0 | 0.96 | 264.36 | High | low_risk | negative |
| CHSGS149 | PAC | 1 | 2 | 72.35 | 2 | 1.72 | 136.15 | High | medium_risk | negative |
| CHSGS150 | Sainfuran | 2 | 5 | 79.91 | 3 | 3.31 | 286.28 | High | medium_risk | negative |
| CHSGS151 | cis-2-Undecenal | 0 | 1 | 47.07 | 8 | 4.93 | 168.28 | High | low_risk | negative |
| CHSGS152 | (+)-Anomalin | 0 | 7 | 46.06 | 6 | 4.67 | 426.46 | High | medium_risk | negative |
| CHSGS153 | cedrenol | 1 | 1 | 108.56 | 0 | 3.64 | 220.35 | High | low_risk | negative |
| CHSGS154 | Ethyl protocatechuate | 2 | 4 | 35.77 | 3 | 1.83 | 182.17 | High | low_risk | negative |
| CHSGS155 | cis-pinocampheol | 1 | 1 | 53.92 | 0 | 2.22 | 154.25 | High | low_risk | negative |
| CHSGS156 | ISOPULEGOL | 1 | 1 | 50.72 | 1 | 2.69 | 154.25 | High | low_risk | negative |
| CHSGS157 | ledol | 1 | 1 | 82.78 | 0 | 3.15 | 222.37 | High | low_risk | negative |
| CHSGS158 | Limetin | 0 | 4 | 36.63 | 2 | 1.91 | 206.19 | High | low_risk | negative |
| CHSGS159 | cis-p-2-Menthen-1-ol | 1 | 1 | 35.3 | 1 | 2.71 | 154.25 | High | low_risk | negative |
| CHSGS160 | (E)-non-2-en-4-one | 0 | 1 | 37.78 | 5 | 2.98 | 140.22 | High | low_risk | negative |
| CHSGS161 | Cumic acid | 1 | 2 | 45.78 | 2 | 2.86 | 164.2 | High | low_risk | negative |
| CHSGS162 | tau-cadinol | 1 | 1 | 36.51 | 1 | 3.52 | 222.37 | High | low_risk | negative |
| CHSGS163 | DTR | 4 | 3 | 75.63 | 3 | -1.1 | 204.23 | High | medium_risk | negative |
| CHSGS164 | Veratryl alcohol | 1 | 3 | 71.49 | 3 | 1.27 | 168.19 | High | low_risk | negative |
| CHSGS165 | β-oplopenone | 0 | 1 | 42.08 | 2 | 3.2 | 220.35 | High | low_risk | negative |
| CHSGS166 | (5S)-5-propyloxolan-2-one | 0 | 2 | 78.49 | 2 | 1.42 | 128.17 | High | low_risk | negative |
| CHSGS167 | Marmesine | 1 | 4 | 84.77 | 1 | 2.1 | 246.26 | High | low_risk | negative |
| CHSGS168 | glyasperin B | 3 | 6 | 65.22 | 4 | 3.14 | 370.4 | High | medium_risk | negative |
| CHSGS169 | glyasperin F | 3 | 6 | 75.84 | 1 | 3.52 | 354.35 | High | medium_risk | negative |
| CHSGS170 | Isotrifoliol | 2 | 6 | 31.94 | 1 | 2.54 | 298.25 | High | medium_risk | negative |
| CHSGS171 | (E)-1-(2,4-dihydroxyphenyl)-3-(2,2-dimethylchromen-6-yl)prop-2-en-1-one | 2 | 4 | 39.62 | 3 | 4.46 | 322.35 | High | medium_risk | negative |
| CHSGS172 | kanzonols W | 2 | 5 | 50.48 | 1 | 3.97 | 336.34 | High | medium_risk | negative |
| CHSGS173 | (2S)-6-(2,4-dihydroxyphenyl)-2-(2-hydroxypropan-2-yl)-4-methoxy-2,3-dihydrofuro[3,2-g]chromen-7-one | 3 | 7 | 60.25 | 3 | 2.61 | 384.38 | High | medium_risk | negative |
| CHSGS174 | Semilicoisoflavone B | 3 | 6 | 48.78 | 1 | 3.55 | 352.34 | High | medium_risk | negative |
| CHSGS175 | Glepidotin A | 3 | 5 | 44.72 | 3 | 2.88 | 338.35 | High | medium_risk | negative |
| CHSGS176 | Glepidotin B | 3 | 5 | 64.46 | 3 | 2.88 | 340.37 | High | medium_risk | negative |
| CHSGS177 | Karenzu DK2 | 0 | 2 | 62.26 | 4 | 3.14 | 224.25 | High | medium_risk | negative |
| CHSGS178 | 8-(6-hydroxy-2-benzofuranyl)-2,2-dimethyl-5-chromenol | 2 | 4 | 58.44 | 1 | 4.27 | 308.33 | High | medium_risk | negative |
| CHSGS179 | Licoricone | 2 | 6 | 63.58 | 5 | 3.08 | 382.41 | High | medium_risk | negative |
| CHSGS180 | Gancaonin A | 2 | 5 | 51.08 | 4 | 3.34 | 352.38 | High | medium_risk | negative |
| CHSGS181 | Gancaonin B | 3 | 6 | 48.79 | 4 | 3.14 | 368.38 | High | medium_risk | negative |
| CHSGS182 | 3-(3,4-dihydroxyphenyl)-5,7-dihydroxy-8-(3-methylbut-2-enyl)chromone | 4 | 6 | 66.37 | 3 | 3.02 | 354.35 | High | medium_risk | negative |
| CHSGS183 | 5,7-dihydroxy-3-(4-methoxyphenyl)-8-(3-methylbut-2-enyl)chromone | 2 | 5 | 30.49 | 4 | 3.29 | 352.38 | High | medium_risk | negative |
| CHSGS184 | 2-(3,4-dihydroxyphenyl)-5,7-dihydroxy-6-(3-methylbut-2-enyl)chromone | 4 | 6 | 44.15 | 3 | 2.99 | 354.35 | High | medium_risk | negative |
| CHSGS185 | Glycyrin | 2 | 6 | 52.61 | 5 | 3.78 | 382.41 | High | medium_risk | negative |
| CHSGS186 | 5,6,7,8-Tetrahydro-2,4-dimethylquinoline | 0 | 1 | 49.77 | 0 | 3.32 | 161.24 | High | medium_risk | negative |
| CHSGS187 | Licocoumarone | 3 | 5 | 33.21 | 4 | 4.1 | 340.37 | High | medium_risk | negative |
| CHSGS188 | Licoisoflavone | 4 | 6 | 41.61 | 3 | 2.99 | 354.35 | High | medium_risk | negative |
| CHSGS189 | Licoisoflavone B | 3 | 6 | 38.93 | 1 | 3.54 | 352.34 | High | medium_risk | negative |
| CHSGS190 | licoisoflavanone | 3 | 6 | 52.47 | 1 | 3.54 | 354.35 | High | medium_risk | negative |
| CHSGS191 | shinpterocarpin | 1 | 4 | 80.3 | 0 | 4.13 | 322.35 | High | medium_risk | negative |
| CHSGS192 | (E)-3-[3,4-dihydroxy-5-(3-methylbut-2-enyl)phenyl]-1-(2,4-dihydroxyphenyl)prop-2-en-1-one | 4 | 5 | 46.27 | 5 | 3.47 | 340.37 | High | medium_risk | negative |
| CHSGS193 | licopyranocoumarin | 3 | 7 | 80.36 | 3 | 2.47 | 384.38 | High | medium_risk | negative |
| CHSGS194 | Glyzaglabrin | 2 | 6 | 61.07 | 1 | 2.32 | 298.25 | High | medium_risk | negative |
| CHSGS195 | Glabridin | 2 | 4 | 53.25 | 1 | 3.81 | 324.37 | High | medium_risk | negative |
| CHSGS196 | Glabranin | 2 | 4 | 52.9 | 3 | 3.59 | 324.37 | High | medium_risk | negative |
| CHSGS197 | Glabrone | 2 | 5 | 52.51 | 1 | 3.78 | 336.34 | High | medium_risk | negative |
| CHSGS198 | 1,3-dihydroxy-9-methoxy-6-benzofurano[3,2-c]chromenone | 2 | 6 | 48.14 | 1 | 2.48 | 298.25 | High | medium_risk | negative |
| CHSGS199 | 1,3-dihydroxy-8,9-dimethoxy-6-benzofurano[3,2-c]chromenone | 2 | 7 | 62.9 | 2 | 2.74 | 328.27 | High | low_risk | negative |
| CHSGS200 | Eurycarpin A | 3 | 5 | 43.28 | 3 | 3.29 | 338.35 | High | medium_risk | negative |
| CHSGS201 | (-)-Medicocarpin | 4 | 9 | 40.99 | 4 | 1.26 | 432.42 | High | medium_risk | negative |
| CHSGS202 | Sigmoidin-B | 4 | 6 | 34.88 | 3 | 3.02 | 356.37 | High | medium_risk | negative |
| CHSGS203 | (2R)-7-hydroxy-2-(4-hydroxyphenyl)chroman-4-one | 2 | 4 | 71.12 | 1 | 2.79 | 256.25 | High | medium_risk | negative |
| CHSGS204 | (2S)-7-hydroxy-2-(4-hydroxyphenyl)-8-(3-methylbut-2-enyl)chroman-4-one | 2 | 4 | 36.57 | 3 | 3.62 | 324.37 | High | medium_risk | negative |
| CHSGS205 | Isoglycyrol | 1 | 6 | 44.7 | 1 | 4.15 | 366.36 | High | low_risk | negative |
| CHSGS206 | HMO | 1 | 4 | 38.37 | 2 | 2.92 | 268.26 | High | medium_risk | negative |
| CHSGS207 | 1-Methoxyphaseollidin | 2 | 5 | 69.98 | 3 | 3.66 | 354.4 | High | medium_risk | negative |
| CHSGS208 | Quercetin der. | 3 | 7 | 46.45 | 3 | 2.55 | 330.29 | High | medium_risk | negative |
| CHSGS209 | (Z)-1-(2,4-dihydroxyphenyl)-3-phenylprop-2-en-1-one | 2 | 3 | 73.18 | 3 | 3.3 | 240.25 | High | medium_risk | negative |
| CHSGS210 | 3'-Hydroxy-4'-O-Methylglabridin | 2 | 5 | 43.71 | 2 | 3.76 | 354.4 | High | medium_risk | negative |
| CHSGS211 | 3'-Methoxyglabridin | 2 | 5 | 46.16 | 2 | 3.76 | 354.4 | High | medium_risk | negative |
| CHSGS212 | Inflacoumarin A | 2 | 4 | 39.71 | 3 | 4.36 | 322.35 | High | medium_risk | negative |
| CHSGS213 | 2,6,10-trimethyl-dodecane | 2 | 4 | 37.8 | 0 | -1.16 | 144.13 | High | low_risk | negative |
| CHSGS214 | 5,6,7,8-Tetrahydro-4-methylquinoline | 0 | 1 | 59.18 | 0 | 2.66 | 147.22 | High | medium_risk | negative |
| CHSGS215 | 6-prenylated eriodictyol | 4 | 6 | 39.22 | 3 | 2.99 | 356.37 | High | medium_risk | negative |
| CHSGS216 | 7,2',4'-trihydroxy－5-methoxy-3－arylcoumarin | 3 | 6 | 83.71 | 2 | 2.51 | 300.26 | High | medium_risk | negative |
| CHSGS217 | 7-Acetoxy-2-methylisoflavone | 0 | 4 | 38.92 | 3 | 3.41 | 294.3 | High | medium_risk | negative |
| CHSGS218 | 8-prenylated eriodictyol | 4 | 6 | 53.79 | 3 | 2.99 | 356.37 | High | medium_risk | negative |
| CHSGS219 | Gancaonin G | 2 | 5 | 60.44 | 4 | 3.25 | 352.38 | High | medium_risk | negative |
| CHSGS220 | Gancaonin H | 3 | 6 | 50.1 | 3 | 3.99 | 420.45 | High | medium_risk | negative |
| CHSGS221 | Licoagrocarpin | 1 | 4 | 58.81 | 3 | 3.94 | 338.4 | High | medium_risk | negative |
| CHSGS222 | Glyasperins M | 2 | 6 | 72.67 | 2 | 3.57 | 368.38 | High | medium_risk | negative |
| CHSGS223 | Glycyrrhiza flavonol A | 4 | 7 | 41.28 | 1 | 2.18 | 370.35 | High | medium_risk | negative |
| CHSGS224 | Licoagroisoflavone | 2 | 5 | 57.28 | 2 | 2.95 | 336.34 | High | medium_risk | negative |
| CHSGS225 | Odoratin | 2 | 6 | 49.95 | 3 | 2.81 | 314.29 | High | medium_risk | negative |
| CHSGS226 | Phaseol | 2 | 5 | 78.77 | 2 | 4.59 | 336.34 | High | medium_risk | negative |
| CHSGS227 | Mipax | 0 | 4 | 57.4 | 4 | 1.96 | 194.18 | High | low_risk | negative |
| CHSGS228 | 5,7-dihydroxy-2-(3-hydroxy-4-methoxyphenyl)chroman-4-one | 3 | 6 | 47.74 | 2 | 2.52 | 302.28 | High | medium_risk | negative |
| CHSGS229 | neobyakangelico l | 1 | 6 | 36.18 | 5 | 2.05 | 316.31 | High | medium_risk | negative |
| CHSGS230 | {5-[2'(R)-Hydroxy-3'-methyl-3'-butenyl-oxy]furocoumarin} | 1 | 5 | 42.85 | 4 | 2.06 | 286.28 | High | medium_risk | negative |
| CHSGS231 | suberosin | 0 | 3 | 53.35 | 3 | 3.46 | 244.29 | High | medium_risk | negative |
| CHSGS232 | Byakangelicol | 0 | 6 | 41.42 | 4 | 2.78 | 316.31 | High | low_risk | negative |
| CHSGS233 | sen-byakangelicol | 0 | 7 | 58 | 6 | 3.99 | 386.4 | High | medium_risk | negative |
| CHSGS234 | Citromitin | 0 | 8 | 86.9 | 7 | 2.61 | 404.41 | High | low_risk | negative |
| CHSGS235 | 2-(2-butynyl)-cyclohexanone | 0 | 1 | 47.78 | 1 | 2.99 | 150.22 | High | low_risk | negative |
| CHSGS236 | 22410-74-8 | 1 | 1 | 39.91 | 4 | 2.9 | 154.25 | High | low_risk | negative |
| CHSGS237 | nobiletin | 0 | 8 | 61.67 | 7 | 2.61 | 402.39 | High | low_risk | negative |
| CHSGS238 | 7-Demethylsuberosin | 1 | 3 | 41.19 | 2 | 3.41 | 230.26 | High | medium_risk | negative |
| CHSGS239 | Methylenetanshinquinone | 0 | 3 | 37.07 | 0 | 3.12 | 278.3 | High | medium_risk | negative |
| CHSGS240 | (-)-beta-Fenchol | 1 | 1 | 79.24 | 0 | 2.3 | 154.25 | High | low_risk | negative |
| CHSGS241 | N-Methyltyramine | 2 | 2 | 75.52 | 3 | 0.48 | 151.21 | High | low_risk | negative |
| CHSGS242 | ZINC00901303 | 0 | 1 | 54.16 | 2 | 1.19 | 86.13 | High | low_risk | negative |
| CHSGS243 | ()-N-Methylephedrine | 1 | 2 | 63.64 | 3 | 1.73 | 179.26 | High | medium_risk | negative |
| CHSGS244 | 2,3-Butanediol, meso- | 2 | 2 | 38.61 | 1 | -0.59 | 90.12 | High | low_risk | negative |
| CHSGS245 | Nerylacetone | 0 | 1 | 45.53 | 6 | 4.59 | 194.31 | High | low_risk | negative |
| CHSGS246 | py | 0 | 1 | 42.32 | 0 | 0.7 | 79.1 | High | medium_risk | negative |
| CHSGS247 | Cubebin | 1 | 6 | 57.13 | 4 | 2.54 | 356.37 | High | medium_risk | negative |
| CHSGS248 | D-synephrine | 3 | 3 | 79 | 3 | -0.62 | 167.21 | High | low_risk | negative |
| CHSGS249 | Marmin | 2 | 5 | 38.23 | 7 | 3.01 | 332.39 | High | medium_risk | negative |
